# Loss of *MEN1* leads to renal fibrosis and decreases HGF‐Adamts5 pathway activity via an epigenetic mechanism

**DOI:** 10.1002/ctm2.982

**Published:** 2022-08-15

**Authors:** Bangming Jin, Jiamei Zhu, Yuxia Zhou, Li Liang, Yunqiao Yang, Lifen Xu, Tuo Zhang, Po Li, Ting Pan, Bing Guo, Tengxiang Chen, Haiyang Li

**Affiliations:** ^1^ Department of Surgery Affiliated Hospital of Guizhou Medical University Guiyang China; ^2^ School of Basic Medical Sciences Guizhou Medical University Guiyang China; ^3^ Transformation Engineering Research Center of Chronic Disease Diagnosis and Treatment Guizhou Medical University Guiyang China; ^4^ Guizhou Provincial Key Laboratory of Pathogenesis and Drug Research on Common Chronic Diseases Guizhou Medical University Guiyang China; ^5^ Guizhou Institute of Precision Medicine Affiliated Hospital of Guizhou Medical University Guiyang China

**Keywords:** epigenetic mechanism, epithelial‐to‐mesenchymal transition (EMT), H3K4me3, *MEN1* gene, renal fibrosis

## Abstract

**Background:**

Renal fibrosis is a serious condition that results in the development of chronic kidney diseases. The MEN1 gene is an epigenetic regulator that encodes the menin protein and its role in kidney tissue remains unclear.

**Methods:**

Kidney histology was examined on paraffin sections stained with hematoxylin‐eosin staining. Masson’s trichrome staining and Sirius red staining were used to analyze renal fibrosis. Gene and protein expression were determined by quantitative real‐time PCR (qPCR) and Western blot, respectively. Immunohistochemistry staining in the kidney tissues from mice or patients was used to evaluate protein levels. Flow cytometry was used to analyze the cell cycle distributions and apoptosis. RNA‐sequencing was performed for differential expression genes in the kidney tissues of the Men1f/f and Men1∆/∆ mice. Chromatin immunoprecipitation sequencing (ChIP‐seq) was carried out for identification of menin‐ and H3K4me3‐enriched regions within the whole genome in the mouse kidney tissue. ChIP‐qPCR assays were performed for occupancy of menin and H3K4me3 at the gene promoter regions. Luciferase reporter assay was used to detect the promoter activity. The exacerbated unilateral ureteral obstruction (UUO) models in the Men1f/f and Men1∆/∆ mice were used to assess the pharmacological effects of rh‐HGF on renal fibrosis.

**Results:**

The expression of MEN1 is reduce in kidney tissues of fibrotic mouse and human diabetic patients and treatment with fibrotic factor results in the downregulation of MEN1 expression in renal tubular epithelial cells (RTECs). Disruption of MEN1 in RTECs leads to high expression of α‐SMA and Collagen 1, whereas MEN1 overexpression restrains epithelial‐to‐mesenchymal transition (EMT) induced by TGF‐β treatment. Conditional knockout of MEN1 resulted in chronic renal fibrosis and UUO‐induced tubulointerstitial fibrosis (TIF), which is associated with an increased induction of EMT, G2/M arrest and JNK signaling. Mechanistically, menin recruits and increases H3K4me3 at the promoter regions of hepatocyte growth factor (HGF) and a disintegrin and metalloproteinase with thrombospondin motifs 5 (Adamts5) genes and enhances their transcriptional activation. In the UUO mice model, exogenous HGF restored the expression of Adamts5 and ameliorated renal fibrosis induced by Men1 deficiency.

**Conclusions:**

These findings demonstrate that MEN1 is an essential antifibrotic factor in renal fibrogenesis and could be a potential target for antifibrotic therapy.

## Graphical abstract

- *MEN1* deficiency leads to chronic renal fibrosis
- *MEN1* deficiency triggers EMT, G2/M arrest and JNK signaling activation
- Hgf‐Adamts5 is a novel target gene that is epigenetically regulated by menin

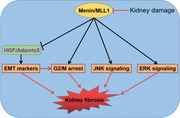

## 1 INTRODUCTION

Kidney fibrosis is a common ultimate pathway in nearly all progressive chronic kidney diseases and is characterized by an excessive deposition of extracellular matrix (ECM), subsequently giving rise to a destruction of the renal parenchyma and advanced kidney failure.^1^ Currently, therapeutic interventions for this devastating condition in the clinical context are almost ineffective. An approved therapeutic option specifically targeting kidney fibrosis is lacking. Under this background, elucidation of the intricate molecular mechanisms underlying kidney fibrosis is crucial adjective. Kidney fibrogenesis is a dynamic and converging process and its pathogenesis engages diverse cellular mechanisms and molecular pathways. These mechanisms, which include inflammatory infiltrate, cell dedifferentiation, senescence, autophagy and metabolic changes, result in the development of progressive fibrotic kidney disease.^2^ As the kidney is a major epithelial organ, defects in functional renal tubular epithelial cells (RTECs) have been recognized as a hallmark of functional decline. Some studies have indicated that RTECs ultimately transdifferentiate into myofibroblasts in the progression of fibrosis via epithelial‐to‐mesenchymal transition (EMT).^3^, ^4^ Recent data have demonstrated that a dysfunction of RTECs plays a pivotal role in the production of ECM during kidney fibrosis^5^ and that this process is governed by the PI3k‐Akt^6^ and p53/CTGF pathways.^7^ However, the molecular mechanism by which tubular epithelial cells initiate renal fibrosis remains unclear.

Menin is an epigenetic regulator encoded by the *MEN1* gene that is associated with multiple endocrine neoplasia type 1 (MEN1).^8^ Menin is a nuclear protein that has no intrinsic enzymatic activity and is preferentially expressed in the brain, thymus and liver at a later gestational stage.^9^ At 20 weeks of age, menin is broadly expressed in many adult tissues, including the brain cortex, adrenal gland, pituitary gland, heart, kidney, testis and thyroid,^10^ implying that *MEN1* expression extends well beyond the tissues involved in *MEN1* pathology and suggesting that it may have important biological functions in these organs. Although *MEN1* is ubiquitous in a variety of organs during mouse embryonic development, its function is tissue‐specific and sometimes exhibits opposing effects across different organs. *MEN1* was originally identified as a tumour suppressor, and mutations of the *MEN1* in humans have been associated with various endocrine tumours, including parathyroid hyperplasia, pituitary adenomas and islet cell tumours.^11^, ^12^ *MEN1* deficiency also induces gastrointestinal stromal, prostate and lung tumour.^13^, ^14^, ^15^ In contrast, menin promotes hepatocellular carcinogenesis by epigenetically upregulating Yap1 transcription.^16^ It has been reported that *MEN1* has dual roles in tumour suppression and proliferation in breast tumourigenesis.^17^, ^18^ Menin interacts with a mixed lineage leukaemia protein 1 (MLL1), which have methyltransferase activity for trimrthylation of lysine 4 of histone H3 (H3K4me3), activating the transcription of multiple targeted genes.^19^ In terms of fibrotic disorders, an early study suggested that menin is a pivotal regulator of activated signalling networks in hepatic fibrogenesis.^20^ A recent study demonstrated that the levels of menin by degrees diminish with the progression of fibrosis in a mouse model of radiation‐induced pulmonary fibrosis.^21^ The potential link between *MEN1* expression and fibrosis provides a novel chance to reveal the biological function of *MEN1* in renal fibrogenesis.

In this work, we show that loss of *Men1* leads to chronic renal fibrosis and exacerbates unilateral ureteral obstruction (UUO)‐induced tubulointerstitial fibrosis (TIF). In an in vitro experiment, we found that *MEN1* deficiency triggers EMT, G2/M arrest and JNK signalling activation. We unravelled a novel epigenetic mechanism involving menin‐mediated regulation of the hepatocyte growth factor (HGF)‐Adamts5 pathway in the progression of kidney fibrosis. Remarkably, recombinant human HGF restored the expression of Adamts5 and ameliorated UUO‐induced kidney fibrosis in the *Men1* knockout (KO) mice. Our findings underscore the significance of preserving *MEN1* expression in the kidney as a therapeutic strategy to delay the progression of renal fibrogenesis.

## 2 MATERIALS AND METHODS

### 2.1 Cell culture

HK‐2 cells (human RTECs) were maintained in DMEM/F12 medium (Gibco) supplemented with 10% fetal bovine serum (FBS), 1% penicillin and 1% streptomycin. NRK‐52E cells (rat RTECs) were maintained in 1‐g/L low glucose DMEM medium (Gibco) supplemented with 10% FBS, 4‐mM l‐glutamine, and 110‐mg/L sodium pyruvate, 1% penicillin and 1% streptomycin. All cells were cultured at 37°C in an atmosphere of 5% CO_2_. For cell cycle analysis, cells were synchronized in serum‐free medium for 24 h and treated separately with 10‐ng/ml TGF‐β (CA59, Novoprotein), 10‐ng/ml IL‐1β (CG99, Novoprotein), 10‐μM MI‐3 (HY‐15223, MCE) or 5‐μg/ml aristolochic acid (AA) (MCE, HY‐N0511) for different periods of time. The collagen 1α (ab210579, Abcam) and TGF‐β1 (ml057830, Mlbio) contents were detected by using the enzyme‐linked immunosorbent assay (ELISA) kits following the manufacturer's instructions.

### 2.2 Animals

Eight‐week‐old C57BL/KS *db*/*db* (Lepr‐KO, *n* = 27) and normal control (NC) C57BL/KS mice (Lepr‐WT, *n* = 20) were purchased from GemPharmatech Co., Ltd (Beijing, China). The mice were killed at weeks 16 and 40, and kidney and blood samples were collected for follow‐up experiments. All experimental *db*/*db* mice, but not the NC mice, display spontaneously diabetic symptoms, such as the increased blood glucose and serum creatinine levels. Diabetic mice were further confirmed by electron microscopy and periodic acid–Schiff staining. The whole‐body expressed *Ubc‐Cre* recombinase mice were crossed with mice harbouring floxed alleles of *Men1* to generate *Men1* ^flox/flox^ (*Men1* ^f/f^);*Ubc‐Cre* mice. Homozygous floxed *Men1* ^f/f^ mice without Cre were considered controls. Dissolved tamoxifen (TAM) in corn oil contains 10% ethanol. At 4–6 weeks of age, the *Men1* ^f/f^;*Ubc‐Cre* and *Men1* ^f/f^ mice received intraperitoneal (*i.p*.) of 100‐mg/kg TAM (T5648‐1G, Sigma) once daily for 5 days to establish whole‐body *Men1* KO (*Men1* ^∆/∆^) mice. Mice were killed at 1, 4, 8 and 12 months after TAM injection and RT‐PCR was used to verify the genotype (*n* = 6 mice in the *Men1* ^f/f^ and *Men1* ^Δ/Δ^ groups at 1 and 4 months; *n* = 9 mice in the *Men1* ^f/f^ and *Men1* ^Δ/Δ^ groups at 8 months; *n* = 9 mice in the *Men1* ^f/f^ and *n* = 12 mice in the *Men1* ^Δ/Δ^ groups at 12 months). All mice were housed under standard conditions with a light/dark cycle of 12 h and free access to food and water. Laboratory conditions were maintained at a constant temperature of 22–25°C and relative humidity of 50%–60%.

Kidney histology was examined on paraffin sections stained with haematoxylin–eosin (H&E) staining. Images were captured by an Olympus VS200 SLIDEVIEW microscopy, and three serial sections were used to analyse kidney injury score. Histologic analyses of H&E‐stained slides were scored, including podocyte hypertrophy and hyperplasia, glomerulosclerosis index and tubulointerstitial changes as previously described.^22^ The degree of interstitial fibrosis was scored 4 grades: 0, no lesions; 1, <25% of parenchyma affected by lesions; 2, 25%–50% of parenchyma affected by lesions; 3, 50%–75% of parenchyma affected by lesions; 4, >75% of parenchyma affected by lesions, as previously described.^23^ The levels of blood glucose were measured using a glucometer (Yuwell720, China). The concentrations of creatinine (C011‐2‐1, Nanjing Jiancheng Bioengineering Institute), collagen 1α and TGF‐β1 were measured by ELISA kits following the manufacturer's instructions.

### 2.3 Masson's trichrome staining and Sirius red staining

Masson's trichrome staining (G1340, Solarbio) and Sirius red staining (DC0041, Solarbio) were carried out according to the kit's instructions, respectively. Images were captured by an Olympus VS200 SLIDEVIEW microscopy and ImageJ software was used to quantify the percentage of the Masson's trichrome and Sirius red positive staining.

### 2.4 Isolation and immortalization of primary mouse renal tubular epithelial cells (mRTECs)

Kidney cortices from 8‐week‐old mice were excised, minced and digested in .5‐mg/ml collagenase (9001‐12‐1, Sigma) for 30 min at 37°C. After digestion, the cortical suspensions were filtered through a 200‐mesh followed by 325‐mesh filters then resuspended in 45% Percoll and centrifuged at 25 000 × *g* for 30 min to separate into four distinct layers. The layer enriched in proximal tubular segments was removed, centrifuged and then resuspended in culture media. The genotypes of *Men1* ^f/f^ and *Men1* ^f/f^;*Ubc*‐*Cre* RTECs were identified by PCR. The mouse RTECs (mRTECs) were induced with 1‐μM TAM to generate primary *Men1* ^f/f^ and Men1^Δ/Δ^ RTECs, named passage 1 (P1) cells. The *Men1* ^f/f^ and *Men1* ^Δ/Δ^ RTECs at P2 were seeded, infected twice with pGMLV‐SV40T lentiviral particles and subculture to established immortalized RTECs. Subsequently, the cells were induced with 1‐μM TAM to establish stable immortalized *Men1* ^f/f^ and Men1^Δ/Δ^ RTEC lines, which were used for subsequent experiments unless otherwise specified. The mRTECs were maintained in 1‐g/L low glucose DMEM medium (Gibco) supplemented with 10% FBS, 4‐mM l‐glutamine and 110‐mg/L sodium pyruvate, 1% penicillin and 1% streptomycin.

### 2.5 Human kidney samples

The patients were diagnosed diabetic nephropathy (DN) with minimal change diseases (MCD) (*n* = 9), stage II (*n* = 8), stage III (*n* = 8) and stage IV (*n* = 10) by two experienced pathologists at the Affiliated Hospital of Guizhou Medical University. MCD: (1) optical microscopy diagnosed as patients with clear MCD; (2) electron microscopy diagnosed as glomerular basement membrane (GBM) was thickened (GBM was >395 nm in female and >430 nm in male). Stage II: (1) optical microscopy diagnosed as patients with mild mesangial hyperplasia; (2) GBM was obvious thickened diagnosed with electron microscopy. Stage III: (1) patients presented with one or more glomerular tuberous sclerosis diagnosed with optical microscopy; (2) electron microscopy diagnosed as mesangial matrix increased and GBM thickened evidently. Stage IV: patients with advanced diabetic glomerulosclerosis, microscopically, more than 50% of the glomerulus displays glomerulosclerosis, and pathologic symptoms similar to those in grade III DN patients further worsened. Human subject characteristics for MCD and DN samples are given in Table 1.

**TABLE 1:** The basic clinical information of minimal change diseases (MCD) and diabetic nephropathy (DN) patients

Characteristics	MCD (*n* = 9)	DN‐II (*n* = 8)	DN‐III (*n* = 8)	DN‐IV (*n* = 10)
Age (years) (mean) [SD]	34.22 (22.69)	61.0 (5.90)	55.5 (8.93)	47.3 (10.45)
Gender				
Male	4	4	8	5
Female	5	4	0	5
BMI (mean) [SD]	23.9 (3.74)	24.26 (2.83)	27.15 (3.25)	25.06 (4.33)
Triglyceride (mM) (mean) [SD]	3.34 (1.29)	2.01 (.66)*	1.54 (.82)*	3.63 (4.03)
Cholesterol (mM) (mean) [SD]	11.7 (2.78)	4.62 (1.01)*	4.23 (1.03)*	5.79 (2.72)*
High‐density lipoprotein (mM) (mean) [SD]	2.07 (.70)	1.34 (.35)*	1.08 (.15)*	1.05 (.37)*
Low‐density lipoprotein (mM) (mean) [SD]	7.42 (1.70)	2.99 (.77)*	2.66 (.79)*	3.77 (1.91)*
Serum creatinine (mM) (mean) [SD]	68.8 (17.22)	81.56 (30.95)	172.05 (84.30)*	418.64 (157.78)*
Blood urea nitrogen (mM) (mean) [SD]	6.45 (2.37)	5.67 (1.88)	10.78 (4.61)*	17.55 (10.69)*
Urine protein (mM) (mean) [SEM]	2077.77 (389.87)	3250.30 (750.48)	4554.01 (753.46)*	5469.67 (552.09)*

*Note*: One‐way ANOVA, compared to MCD patients.

Abbreviation: SD, standard deviation.

^*^ *p* < .05.

### 2.6 Western blotting

Protein lysates from kidneys or cells were conducted SDS polyacrylamide gels (SDS–PAGE) and transferred to PVDF membranes, and then to Western blot to determine the expression of indicated proteins following the standard procedure. The primary antibodies were adopted: menin (A300‐105A, Bethyl Laboratories, 1:5000), MLL1 (14197, Cell Signaling Technology, 1:1000), H3K4me3 (17‐614, Millipore, 1:4000), H3K4me2 (07‐030, Millipore, 1:4000), H3K4me1 (07‐436, Millipore, 1:5000), H3K9me3 (ab8898, Abcam, 1:4000), Histone 3 (ab1791, Abcam, 1:20000), α‐SMA (ab5694, Abcam, 1:1000), vimentin (Sc‐6260, Santa Cruz, 1:1000), collagen 1 (ab6586, Abcam, 1:1000), E‐cadherin (3195, Cell Signaling Technology, 1:1000), Kim‐1 (ab47635, Abcam, 1:200), N‐cadherin (610921, BD Biosciences, 1:1000), desmin (ab1466‐1, Abcam, 1:1000), cyclin D1 (Sc‐8396, Santa Cruz, 1:500), Hgf (26881‐1‐AP, Proteintech, 1:1000), Adamts5 (A2836, ABclonal, 1:1000), cyclin B1 (55004‐1‐AP, Proteintech, 1:1000), phosoho‐JNK (Thr183/Thr185) (9255S, Cell Signaling Technology, 1:1000), phosoho‐c‐jun (Ser63) (ab32385, Abcam, 1:1000), ATM (ab78, Abcam, 1:1000), phosoho‐ATM (Thr1981) (ab81292, Abcam, 1:1000), phosoho‐ERK (extracellular signal‐regulated kinase) (Thr202/Tyr204) (4376, Cell Signaling Technology, 1:1000), CHK2 (2662S, Cell Signaling Technology, 1:1000) and phosoho‐CHK2 (Thr68) (ab3501, Abcam, 1:1000). Protein bands were visualized using the Tanon Imaging System (Tanon‐5200, Tanon Science Technology) and densitometry was performed by ImageJ software.

### 2.7 Targeted knockdown (KD) of genes with siRNA or shRNA

siRNAs specifically targeting *MEN1* and *HGF* were synthesized by Sangon Biotech, and the sequences are shown in Table S1. HK‐2 cells were transfected with 50‐nM siRNA using Chemi‐Trans FectinBor DNA Transfection Reagent according to the manufacturer's instructions (T008, Gene Codex). For stable HK‐2 cell clones with *MEN1*, retroviral packaging GP2‐293 cells were transfected with sh*MEN1* retroviral plasmids using Chemi‐Trans FectinBor DNA Transfection Reagent, and the retroviruses were collected 48 h after transfection. HK‐2 cells were infected with sh*MEN1* retrovirus particles in the presence of 10‐μg/ml polybrene and selected with 1‐μg/ml puromycin for 2 weeks. Cells infected with retroviruses expressing sh*Luciferase* (sh*Luc*) plasmids were used as controls. To produce *MEN1*‐overexpressing cell lines, HK‐2 cells were infected with retrovirus particles expressing pLNCX2‐*MEN1* plasmids in the presence of 10‐μg/ml polybrene and selected with 1‐mg/ml G418 for 14 days. Cells infected with retroviruses expressing pLNCX2 plasmids were used as controls.

### 2.8 CRISPR/Cas9‐mediated *MEN1* gene knockout

*MEN1*‐deficient HK‐2 cells were generated using CRISPR/Cas9 system, as previously described.^24^ A single‐guide RNA sequence (5′‐GGCACCAAATTGGACAGCTCCGG‐3′) was used for disrupting the expression of *MEN1* in HK‐2 cells. *MEN1* KO cell lines were generated as described.^24^ The disruption of *MEN1* gene was verified by genomic DNA sequencing and Western blot. The *MEN1*‐WT and *MEN1*‐KO HK‐2 cells were exposed to 5‐μg/ml AA for 72 h, conditioned medium (CM) was harvested and added to the fibroblasts culture.

### 2.9 Quantitative real‐time PCR (qPCR)

RNA preparation was performed according to TRIzol reagent, cDNA synthesis was performed according to reverse transcription kit's instructions (RR820A, Takara), and quantitative real‐time PCR (qPCR) was carried out using a BIO‐RAD CFX96 Real‐Time system with the primers listed in Table S1. qPCR was repeated at least three times and gene expressions were calculated based on the 2^−∆∆^ *^Ct^* method.

### 2.10 Immunohistochemistry staining

Kidney tissues were fixed in 4% paraformaldehyde solution, embedded in paraffin and sectioned (5 μm) onto glass slides. Immunohistochemistry was carried out as previously described^25^ by using primary antibodies against menin (A300‐105A, Bethyl Laboratories, 1:2000), H3K4me3 (17‐614, Millipore, 1:1000), H3K4me2 (07‐030, Millipore, 1:500), H3K9me3 (ab8898, Abcam, 1:500), α‐SMA (ab5694, Abcam, 1:200), collagen 1 (ab6586, Abcam, 1:500), E‐cadherin (3195, Cell Signaling Technology, 1:1000), vimentin (Sc‐6260, Santa Cruz, 1:200), N‐cadherin (610921, BD Biosciences, 1:200) and Adamts5 (A2836, ABclonal, 1:200). Negative controls were treated identically, but without primary antibody. Images were captured with a panoramic scan using an Olympus VS200 SLIDEVIEW microscope. Quantification of immunohistochemistry (IHC) staining was carried out by calculating the ratio of the positive staining area to the whole area using Image‐Pro Plus software.

### 2.11 Immunofluorescence staining

Immunofluorescence (IF) staining was performed as previously described.^25^ The primary antibodies were adopted: menin (A300‐105A, Bethyl Laboratories, 1:200), H3K4me3 (17‐614, Millipore, 1:200), F‐actin (ab205, Abcam, 1:100), phosoho‐H3 (Ser10) (ab5176, Abcam, 1:200). Images were captured with a confocal microscope (Olympus SpinSR10).

### 2.12 Flow cytometry

Propidium iodide (PI) staining was performed according to the cell cycle detection kit instructions (KGA512). Briefly, treated cells were washed with ice‐cold PBS and harvested by centrifugation at 1000 × *g* for 5 min. Add ice‐cold 70% ethanol to fix at 4°C overnight. Cells were washed with PBS and treated with 20‐mg/ml RNase A for 30 min. Cells were stained with PI solution for 15 min at room temperature in the dark before cell cycle analysis by flow cytometer (ACEA NovoCyte Fluidics Station, BD Biosciences).

### 2.13 RNA‐sequencing (RNA‐seq)

Total RNA was isolated from the kidney tissues of the *Men1* ^f/f^ and *Men1* ^∆/∆^ mice using TRIzol reagent and purified using poly‐T oligo‐attached magnetic beads. Fragmentation at high temperature using divalent cations in first‐strand synthesis reaction buffers (5×). First‐strand cDNA was synthesized using random hexamer primers and M‐MuLV Reverse Transcriptase. Subsequently, second‐strand cDNA synthesis using DNA polymerase I and RNase H. The 3′ end of the DNA fragment was adenylated and ligated to an adaptor with a hairpin loop structure in preparation for hybridization. The library fragments were purified using the AMPure XP system (Beckman Coulter, Beverly, USA) to obtain cDNA fragments with a length of 370–420 bp. The PCR was carried out with Phusion High‐Fidelity DNA Polymerase, universal PCR primers and Index primers and library quality was assessed using the Agilent Bioanalyzer 2100 system. Six RNA‐sequencing (RNA‐seq) libraries were sequenced on the Illumina NextSeq platform with an average depth of approximately 30 million, 150 nucleotide paired‐end reads per sample. Reads were then aligned using CASSAVA, of which >65% mapping to the reference mouse genome. Reads were aligned to the mouse reference genome using Hisat2 v2.0.5, and the read numbers mapped to each gene were counted using FeatureCounts v1.5.0‐p3. The FPKM of each gene was calculated from the length of the gene and the read counts mapped to that gene. Differential expression analysis was carried out between the *Men1* ^f/f^ and *Men1* ^∆/∆^ mice using DESeq2, and *p* values of <.05 were considered differentially expressed.

### 2.14 Chromatin immunoprecipitation (ChIP)‐sequencing (ChIP‐seq)

For the identification of menin‐ and H3K4me3‐enriched regions within the whole genome, the chromatin immunoprecipitation‐sequencing (ChIP‐seq) analysis was carried out by KangChen Bio‐tech Co., Ltd (Shanghai, China). Twelve‐month‐old mouse kidney tissues were crosslinked with 4% formaldehyde and sonicated to shear chromatin into appropriate fragments. Sequencing libraries were prepared using the TruSeq Nano DNA Sample Prep Kit (Illumina) and 300‐cycle sequencing on the Illumina HiSeq 4000 System using the HiSeq 3000/4000 SBS Kit. MACS v1.4.2 software was run with the mapped reads to detect the statistically significant ChIP‐enriched peaks compared to the corresponding input group with a *p* value threshold of 10^−4^. The differentially enriched peaks were identified by a fold change (FC) >2.0 and *p* value <.001. All regions were annotated by the gene whose transcriptional start sites (TSS) were closest to the centre of the peak region and divided into five categories according to the distance from the UCSC RefSeq genes. Gene visualization was carried out by Integrative Genomics Viewer.

### 2.15 ChIP‐qPCR

ChIP assays were performed with the menin and H3K4me3 antibodies according to the Simple ChIP Kit's protocol (91820S, Cell Signaling Technology). Briefly, 1 × 10^6^ RTECs were crosslinked with 1% formaldehyde for 10 min. Cell pellets were incubated in Buffer A for 10 min, and pellet nuclei were harvested by centrifugation at 2000 × *g* and digested in Buffer B containing 25 units micrococcal nuclease per IP for 20 min at 37°C, followed by pulsed ultrasonication to shear cellular DNA and cleared by centrifugation at 12 000 × *g* for 10 min. Equal amounts of chromatin were incubated overnight at 4°C with primary antibody. The following antibodies were adopted: menin (ab31902, Abcam, 4 μg/IP), H3K4me3 (17‐614, Millipore, 4 μg/IP). DNA pulled down by the antibodies was purified by spin columns and purified DNA was quantitated by qPCR using CFX Connect Real‐time system (CFX Conne, Bio‐Rad).

### 2.16 Luciferase reporter analyses

HK‐2 cells were transfected with the Adamts5 promoter luciferase reporter vector (508 bp, GenePharma, Shanghai, China) using Chemi‐Trans FectinBor DNA transfection reagent according to the manufacturer's instructions. Transfection with pRL *Renilla* luciferase reporter vector was used for normalization and to assess transfection efficiency. Cells were harvested 48 h after transfection and lysed by the addition of passive lysis buffer. Then, the reporter activity was measured using the Dual‐Glo Luciferase Assay System (E2920, Promega). For each sample well, the emission of each firefly luciferase was normalized to that of *Renilla*. Each reaction was carried out in triplicate and in three independent experiments.

### 2.17 In vivo treatment with recombinant human HGF (rh‐HGF)

The *Men1* ^f/f^ (*n* = 18) and *Men1* ^∆/∆^ mice (*n* = 18) were randomly divided into three groups of 6 mice with equal numbers of males and females. The *Men1* ^f/f^ and *Men1* ^∆/∆^ mouse models of renal fibrosis were established by UUO according to an established procedure.^26^ Briefly, 6–8‐week mice were anaesthetized with 5% isoflurane and an incision was made on the outside of the abdomen, and then the left ureter was double ligated with 4‐0 silk thread to establish the UUO models. Ureteral exposure and manipulation in sham‐operated mice is the same as in UUO mice, but without ligation. rh‐HGF (BK0220, Bioword) was dissolved in PBS and treatment started on the day of surgery; the mice were administrated by intravenous injection (*i*.*v*.) at a dose of 200‐μg/kg body weight once a daily for 7 days. The mice that underwent UUO treated with an equal volume of PBS solution served as the vehicle group. All mice were killed at Day 3 after treatment, and kidneys and blood samples were harvested. Some kidneys were fixed in 4% formalin solution for histologic examination and molecular analysis. The remaining kidney tissues were placed at −80°C for protein and mRNA extractions.

### 2.18 Statistical analysis

All data are expressed as the mean and standard deviation and were analysed using the statistical package for the GraphPad Prism 7. Two‐group comparisons were made using two‐tailed Student's *t* tests, and multiple group comparisons were made using one‐way ANOVA analysis of variance. The condition *p* < .05 was considered statistically significant. Each experiment was performed at least in triplicate, unless otherwise indicated.

## 3 RESULTS

### 3.1 Decreased *MEN1* expression in fibrotic kidney disease samples

**FIGURE 1:**
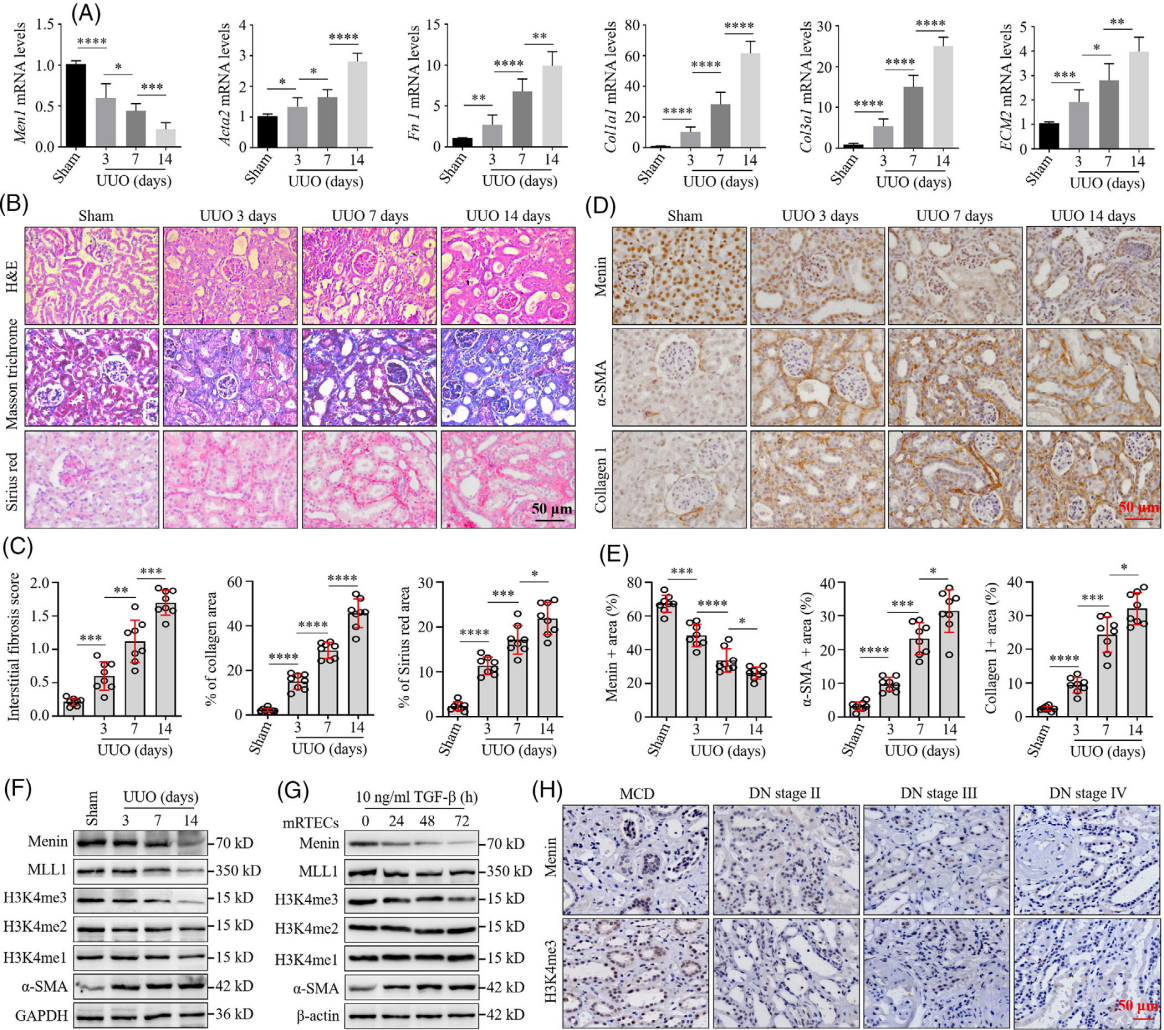
Decreased MEN1 expression in fibrotic kidney disease samples. (A) Quantitative real‐time PCR (qPCR) was used to detect the mRNA expression of Men1, Acta2, Fibronectin1, Col1a1, Col3a1 and ECM2 in the kidney tissues of the sham and unilateral ureteral obstruction (UUO) mice (*n* = 8 mice per group). (B) Representative images of haematoxylin–eosin (H&E), Masson's trichrome and Sirius red staining of kidney sections from the sham and UUO mice; scale bars 50 μm. (C) Quantification of the interstitial fibrosis score, the area of Masson's trichrome and Sirius red staining in the kidney tissues of the sham and UUO mice (*n* = 8 mice per group). (D) Immunohistochemistry (IHC) staining for menin, α‐SMA and collagen 1 in the kidney tissues of the in the kidney tissues of the sham and UUO mice; scale bars 50 μm. (E) Quantification of menin, α‐SMA and collagen 1 IHC staining in (D) (*n* = 8 mice per group). (F) Western blotting was used to detect the expression of the indicated proteins in the kidney tissues of the sham and UUO mice (*n* = 3 mice per group). (G) Western blotting was used to detect the expression of the indicated proteins in mouse renal tubular epithelial cells (mRTECs) at the indicated time points after exposure to 10‐ng/ml TGF‐β. (H) Representative images of menin and H3K4me3 IHC staining in kidney tissues of the minimal change diseases (MCD) and diabetic nephropathy (DN) patients; scale bars 50 μm. The data are represented as the mean ± standard deviation (SD); *p < .05, **p < .01, ***p < .001, ****p < .0001.

To investigate the potential functions of *MEN1* in kidney fibrosis, we first detected the expression pattern of *MEN1* in kidney tissues of the UUO mice, a classical renal TIF model.^27^ As expected, compared to the sham mice, prominent changes in the renal fibrotic phenotype in the UUO mice included a marked upregulation of mRNA expression of *Acta2*, *Fibronectin1* (*Fn1*), *Col1a1*, *Col3a1* and *ECM2* (Figure 1A), an obvious increase in the interstitial fibrosis score, the area of Sirius red and Masson's trichrome staining (Figure 1B,C) and a significant enhancement of α‐smooth muscle actin (α‐SMA) and collagen 1 protein expression (Figure 1D,E). Importantly, the mRNA and protein levels of Men1 in kidney tissues of the UUO mice began to decrease at Day 3 after surgery and subsequently gradually diminished with the progression of the disease (Figure 1A,D,E). Linear regression analysis showed a negative correlation between the levels of *Men1* expression and the levels of *Acta2* expression, as well as the areas of interstitial collagen determined by Masson's trichrome staining (Figure S1A). Similar findings were observed by repeating this experiment in *db*/*db* mice, a well‐studied model of spontaneous DN, that exhibited relatively higher blood glucose, serum creatinine, mRNA expression of fibrogenic markers and interstitial fibrosis when compared to their respective NCs (Figure S1B–E). Western blot results of menin in the kidney tissues of UUO and *db*/*db* mice confirmed the IHC or qPCR findings (Figures 1F and S1F–H). Consistently, IF staining showed a time‐dependent reduction in menin protein expression in the nuclei of HK‐2 cells treated with TGF‐β (Figure S1I,J). TGF‐β or AA induced an obvious decrease of *Men1* mRNA and protein in the mRTECs or NRK‐52E cells (Figures 1G and S1L–N). Our results suggest the dysregulation of the menin expression in renal fibrosis and indicate that this molecule might play a vital role in renal fibrogenesis.

Additionally, we noted that the expression levels of MLL1 (a partner of menin) and H3K4me3 were gradually reduced, but the levels of H3K4me2 and H3K4me1 were not clearly altered in the kidneys of UUO and *db*/*db* mice (Figures 1F and S1F–H). Also, TGF‐β or AA treatment led to a reduction in the expression levels of MLL1 and H3K4me3 in a time‐dependent fashion, whereas the levels of H3K4me2 and H3K4me1 were not clearly affected in both mRTEC and NRK‐52E cells (Figures 1G and S1K–M). The datasets from the Gene Expression Omnibus (GSE121700) showed that *Men1* and *KMT2A* (encoding MLL1) mRNAs in kidney tissues of the C57BL/6J mice treated with TGF‐β were diminished compared with those of the controls (Figure S1O). Finally, we assessed the expression of menin in kidney tissues of human DN. First, we showed the patients’ basic clinical information in Table 1. Furthermore, IHC results demonstrated that the base expression levels of menin and H3K4me3 protein in the patient's kidneys with DN stages II, III and IV were significantly lower than those of the MCD patients and further reduced with a progression of DN (Figures 1H and S1P). Altogether, these data demonstrate that the dysregulation of the menin/MLL1 pathway contributes to the renal fibrogenesis.

### 3.2 Deletion of *Men1* results in progressive kidney damage and fibrosis

**FIGURE 2:**
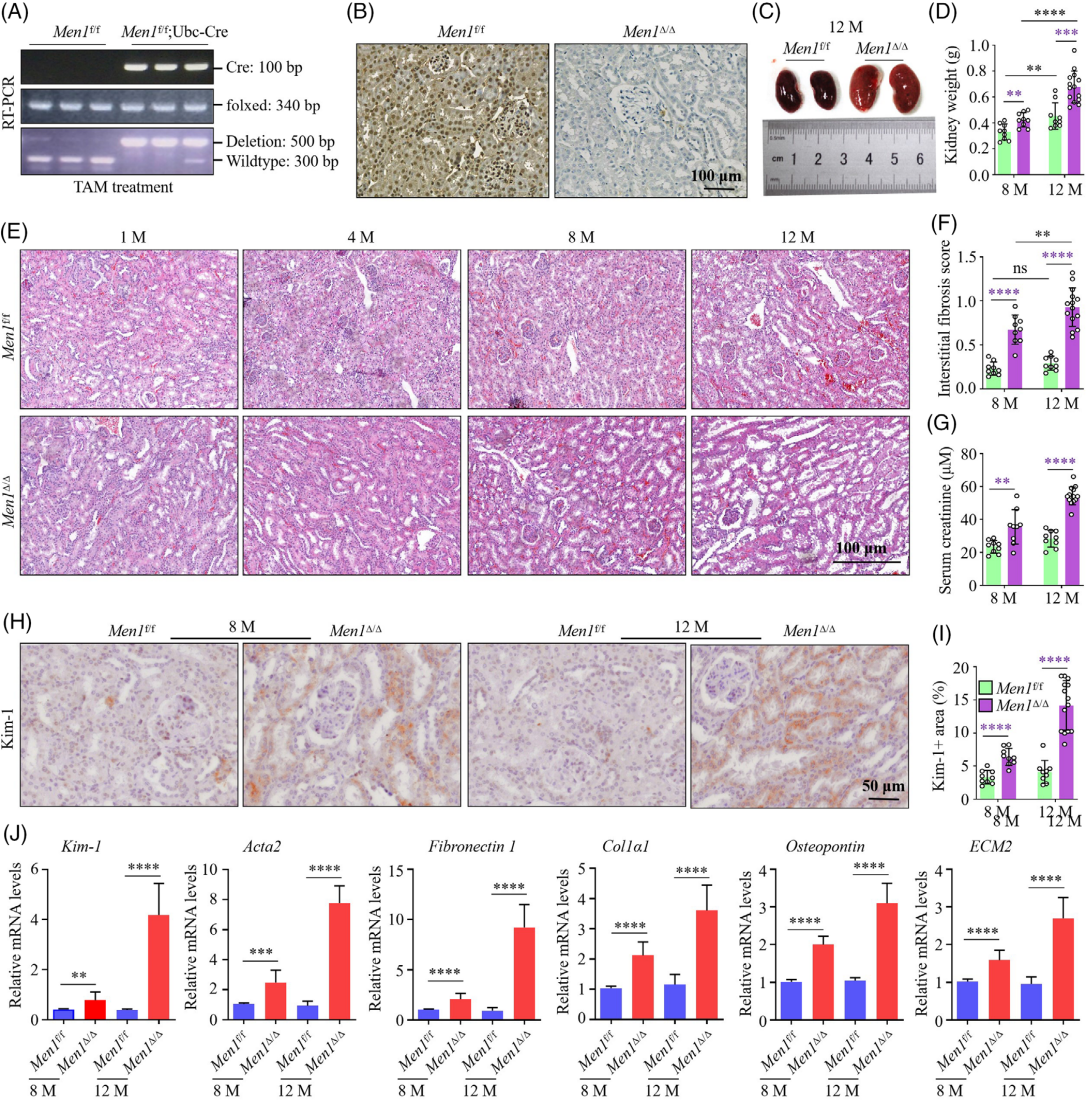
Deletion of Men1 results in progressive kidney damage and fibrosis. (A) PCR was used to identify genotypes in the kidney tissues of the *Men1* ^f/f^ and *Men1* ^f/f^;*Ubc‐Cre* mice 1 week after 100‐mg/kg tamoxifen (TAM) treatment. (B) Representative images of menin immunohistochemistry (IHC) staining of kidney sections from the *Men1* ^f/f^ and Men1^Δ/Δ^ mice at 12 months; scale bars 100 μm. (C) Representative brightfield images of gross kidneys from the *Men1* ^f/f^ and Men1^Δ/Δ^ mice at 12 months. (D) Quantification of kidney weight for the *Men1* ^f/f^ and Men1^Δ/Δ^ mice. (E) Representative haematoxylin–eosin (H&E) images of kidney sections from the *Men1* ^f/f^ and Men1^Δ/Δ^ mice at 1–12 months; scale bars 100 μm. (F) Quantitative analysis of interstitial fibrosis score in the kidney tissues of the *Men1* ^f/f^ and Men1^Δ/Δ^ mice. (G) Enzyme‐linked immunosorbent assays (ELISAs) were used to measure serum creatinine levels of the *Men1* ^f/f^ and Men1^Δ/Δ^ mice. (H) Representative images of Kim‐1 IHC staining of kidney sections from the *Men1* ^f/f^ and Men1^Δ/Δ^ mice at 8 and 12 months; scale bars 100 μm. (I) Quantification of Kim‐1 IHC staining in H. (J) Quantitative real‐time PCR (qPCR) was used to detect the mRNA expression of Kim‐1, Acta2, Fibronectin1, Col1α1 and Osteopontin in the kidney tissues of the *Men1* ^f/f^ and Men1^Δ/Δ^ mice. *n* = 9 mice per group at 8 months; *n* = 9 mice in the *Men1* ^f/f^ and *n* = 14 mice in the Men1^Δ/Δ^ groups at 12 months. The data are represented as the mean ± standard deviation (SD); *p < .05, **p < .01, ***p < .001, ****p < .0001.

We generated a conditional TAM‐inducible *Ubc‐Cre Men1* allele homozygous KO mouse model to elucidate the functional role of *Men1* in the kidney. We crossed *Ubc‐Cre* (a whole‐body expressed recombinase) mice with mice harbouring floxed alleles of *Men1* to generate *Men1* ^f/f^;*Ubc‐Cre* mice. PCR analysis showed that bands of wild‐type or floxed *Men1* alleles were observed at ∼340 and 300 bp, whereas the expression of the *Cre* transgene was visualized by a 100‐bp PCR product (Figure 2A). After conditional TAM induction, the band of *Men1* deletion was detected by a 500‐bp PCR product (Figure 2A), and the KO efficiency of *Men1* in whole kidney tissue of *Men1* ^f/f^;*Ubc‐Cre* mice determined by PCR and IHC, named *Men1* ^Δ/Δ^, was >85% when compared to that of the *Men1* ^f/f^ animals (Figure 2A,B).

After TAM treatment, morphological observation indicated that the normal renal architecture was exhibited in the *Men1* ^Δ/Δ^ mice at 1 month of age. By 4 months, mild kidney oedema started to appear accompanied by local inflammatory cell infiltration in some of the *Men1* ^Δ/Δ^ mice. By 8 months, fibrotic changes began to develop, and most animals presented moderately dilated renal tubules and reduced internal clearance between glomeruli and occasionally cystic appearing. By 12 months, the kidney became severely swollened and the kidney weight obviously enhanced in the *Men1* ^Δ/Δ^ mice when compared with the *Men1* ^f/f^ animals (Figure 2C,D); the normal renal architecture of the *Men1* ^Δ/Δ^ mice was seriously destroyed, the interstitium was widened and kidney tissues emerged the tubular renal tubules (Figure 2E). Histopathological scores showed that loss of *Men1* induced a severe kidney injury relative to the *Men1* ^f/f^ mice and the interstitial fibrosis gradually aggravated over time in the *Men1* ^Δ/Δ^ mice, but not in the *Men1* ^f/f^ animals (Figure 2F and Table 2).

**TABLE 2:** Histologic scoring for kidney sections of *Men1^f/f^* and *Men1* ^Δ/Δ^ mice at 12 months

*Men1* ^f/f^ mice (*n* = 9)	Glomerular collapse/sclerosis	Podocyte hyperplasia (0–3+)	Tubular regenerative changes (0–3+)	Tubular atrophy/interstitial fibrosis (%)
1	1.9% (1/52)	0	0	0
2	3.3% (2/60)	0	0	5
3	7.0% (5/72)	0	0	0
4	3.0% (2/68)	0	0	0
5	4.0% (3/77)	0	0	0
6	.0% (0/61)	0	0	0
7	1.7% (1/58)	0	0	10
8	8.0% (6/75)	0	0	0
9	5.7% (4/70)	0	0	5

*Men1* ^Δ/Δ^ mice (*n* = 14)				
1	51.4% (38/74)	2+	2+	25
2	38.2% (26/68)	1+	1+	20
3	51.5% (33/64)	2+	1+	20
4	43.7% (31/70)	1+	1+	15
5	38.6% (22/57)	1+	2+	25
6	55.6% (40/72)	2+	2+	35
7	45.4% (30/66)	2+	1+	25
8	38.4% (28/73)	1+	1+	15
9	42.6% (23/54)	2+	2+	30
10	28.0% (19/68)	1+	1+	10
11	41.6% (32/77)	1+	2+	15
12	41.3% (26/62)	2+	2+	35
13	41.7% (25/60)	2+	2+	30
14	32.4% (24/78)	1+	2+	20

In‐line with the morphologic alteration, the *Men1* ^Δ/Δ^ mice display severe renal dysfunction and damage, as confirmed by increased the serum creatinine levels and the expression of Kim‐1 (an acute injury marker) in the 8‐ and 12‐month‐old mice compared with the *Men1* ^f/f^ animals (Figure 2G–I). qPCR analysis displayed that the mRNA levels of *Kim‐1* and fibrotic markers such as *Acta2*, *Fibronectin1*, *Col1α1* and *Osteopontin* in the *Men1* ^Δ/Δ^ mice were memorably higher than that of the *Men1* ^f/f^ mice (Figure 2J). Loss of *Men1* predominantly augmented the area of Masson's trichrome and Sirius red staining in the kidney tissues at 8 and 12 months (Figure S2A–C). Similarly, the deletion of *Men1* dramatically promoted the mRNA expression of *Kim‐1*, *Acta2*, *Fibronectin1*, *Col1α1* and *ECM2* in mRTECs with or without TGF‐β in a time‐dependent fashion (Figure S2D), further confirming the severe fibrotic development in the *Men1* ^Δ/Δ^ mice. These findings demonstrate that deficiency of *Men1* gene leads to progressive whole kidney damage and chronic renal fibrosis.

### 3.3 Deletion of *Men1* induces fibrosis‐related changes in signalling pathway networks

**FIGURE 3:**
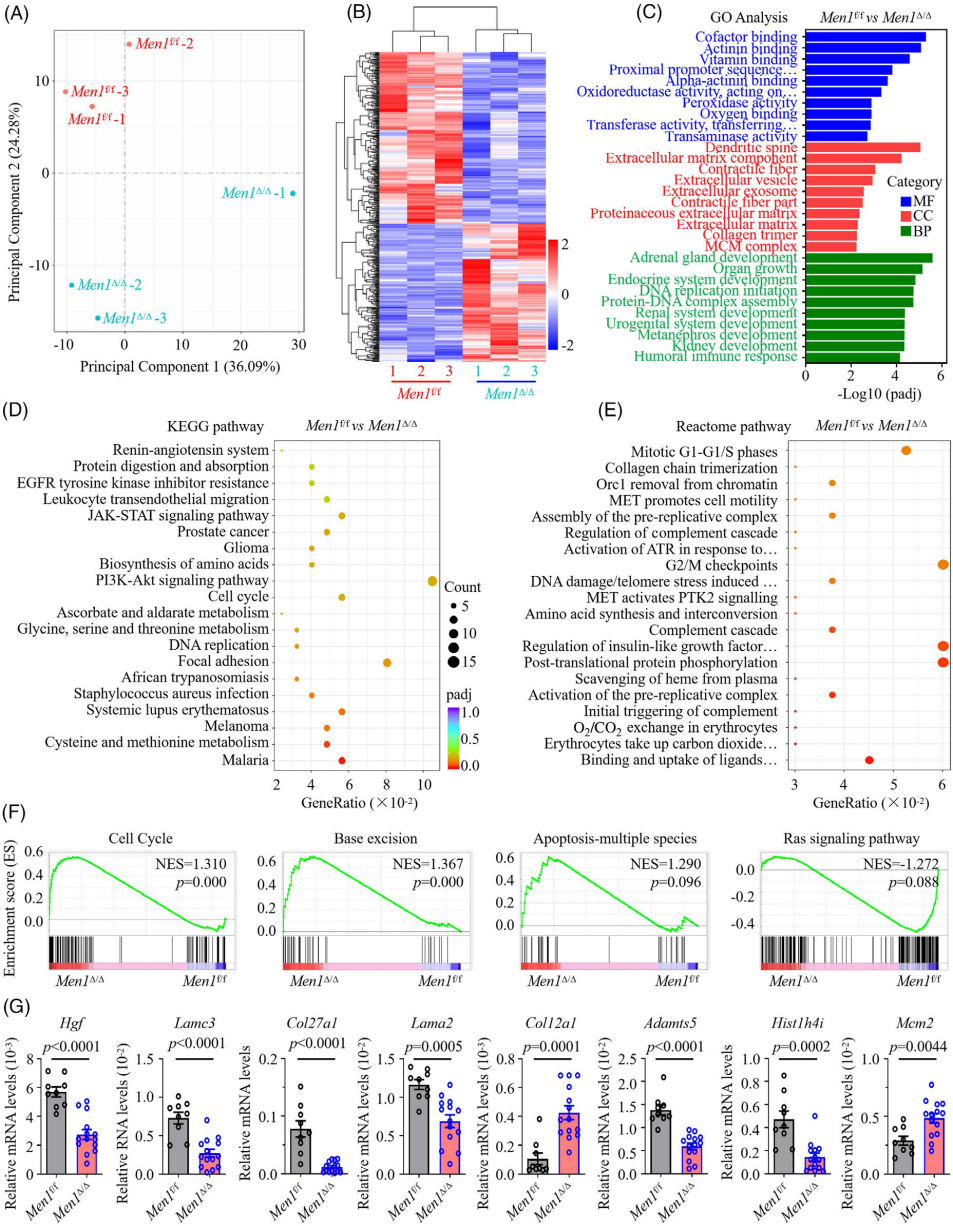
Deletion of Men1 induces fibrosis‐related changes in signalling pathway networks. (A) Multidimensional scaling plot showing separation of the *Men1* ^f/f^ and Men1^Δ/Δ^ mouse kidney tissues at 12 months. (B) Heat map representing transcripts with upregulated (red) or downregulated (blue) expression in kidney tissues of the *Men1* ^f/f^ and Men1^Δ/Δ^ mice. (C) Gene ontology (GO) enrichment analysis showing representative biological processes (BPs), molecular functions (MFs) and cellular components (CCs) for differentially expressed genes between the *Men1* ^f/f^ and Men1^Δ/Δ^ mouse kidney tissues. (D and E) BubbleMap showing representative KEGG and Reactome pathways for fibrosis‐related pathways affected by Men1 deficiency. (F) GSEA of cell cycle, base excision, apoptosis‐multiple species and Ras signalling genes in kidney tissues of the *Men1* ^f/f^ and Men1^Δ/Δ^ mice at 12 months. (G) Quantitative real‐time PCR (qPCR) was used to detect the mRNA expression of the indicated genes in the kidney tissues of the *Men1* ^f/f^ and Men1^Δ/Δ^ mice at 12 months (*n* = 9 mice in the *Men1* ^f/f^ and *n* = 14 mice in the Men1^Δ/Δ^ groups); the data are represented as mean ± standard deviation (SD) (*t*‐test, two‐sided).

To delineate molecular features and potential cellular signalling pathways in the mice with kidney fibrosis, we analysed the gene expression profile in whole kidney tissues from three normal *Men1* ^f/f^ mice and three *Men1* ^Δ/Δ^ mice with kidney fibrosis. We performed two‐dimensional principal component analysis after sample normalization and variance‐stabilizing transformation to ensure that there were no technical batch effects (Figure 3A). Hierarchical clustering analysis revealed that *Men1* ^f/f^ and *Men1* ^Δ/Δ^ mouse replicates in the same group had high reproducibility (Figure 3B). A volcano plot showed that a total of 469 transcripts were differentially expressed in the kidneys of the *Men1* ^Δ/Δ^ mice relative to those of the normal *Men1* ^f/f^ mice, with 208 of these transcripts showing upregulated expression and 261 showing downregulated expression (Figure S3A and Data S1). Among the genes with downregulated expression were nephrosis‐related genes such as *Nphs1* (FC = −2.588, *p* value = 1.17e−7) and *Nphs2* (FC = −2.534, *p* value = 2.17e−12), which are frequently mutated in familial nephrotic syndrome^28^ (Figure S3A and Data S1). Additionally, the transcripts of *Gdf11* (FC = −1.682, *p* value = 1.80e−5), *Adamts5* (FC = −1.508, *p* value = 3.55e−7), *Hgf* (FC = −2.664, *p* value = 1.54 × 10^−4^), *Pdgfrα* (FC = −1.685, *p* value = 2.29e−5) and *Pdgfrβ* (FC = −1.031, *p* value = 2.30 × 10^−4^), which are important regulators involved in the development of renal fibrosis,^29^, ^30^, ^31^, ^32^ were significantly expressed at lower levels in the *Men1* ^Δ/Δ^ mice. In contrast, some profibrogenic genes, such as *Socs2* (FC = 2.0697, *p* value = 5.22e−10), *Cdkn1a* (FC = 1.465, *p* value = 3.22e−5), *Wnt5a* (FC = 1.444, *p* value = 2.07e−6), *Nfil3* (FC = 2.014, *p* value = 5.05e−7) and *Col12a1* (FC = 1.16, *p* value = 2.14 × 10^−4^), showed upregulated expression in the *Men1* ^Δ/Δ^ mice relative to the *Men1* ^f/f^ mice^33^, ^34^, ^35^, ^36^ (Figure S3A and Data S1). These results indicate that *Men1* deficiency significantly contributes to the gene expression profile of renal fibrosis.

Gene ontology (GO) analysis shows that the most significant differentially expressed genes (DEGs) were enriched for biological processes (BPs) related to kidney development, urogenital system development and renal system development and molecular functions (MFs) such as transaminase activity, alpha‐actinin binding and cofactor binding (Figure 3C). Specifically, the most significantly enriched GO cellular components were collagen trimer, ECM, contractile fibre and ECM component (Figure 3C). KEGG and Reactome pathway enrichment analyses demonstrated that the DEGs were involved in signalling pathways closely related to fibrosis, which included the JAK‐STAT signalling pathway,^37^ PI3k‐Akt signalling pathway,^38^ collagen chain trimerization^39^ and G2/M checkpoints^23^ (*p* value <.05, Figure 3D,E). In the *Men1* ^Δ/Δ^ mice, the expression of gene sets in these downregulated pathways was significantly reduced (Figure S3B–D), whereas expression was elevated in upregulated pathways, compared with the that of the *Men1* ^f/f^ mice (Figure S3E,F). The GSEA plot displayed that gene related to the cell cycle, base excision pathway and apoptosis‐multiple species tend to be highly expressed, whereas Ras signalling pathway genes, which induce fibrogenic EMT and intratumoural fibrosis,^40^ were expressed at low levels in the *Men1* ^Δ/Δ^ mice (Figure 3F). These results suggest that *Men1* deficiency gives rise to renal fibrosis via multiple signalling pathways.

To experimentally validate the transcriptomics results, we selected six genes with downregulated expression and two with upregulated expression from the edgeR analysis, and we performed qPCR on the same cohort to determine their expression levels in the kidney tissues of the *Men1* ^f/f^ and *Men1* ^Δ/Δ^ mice. The genes selected for testing have functional annotations involving regulation of MET activation of PTK2 signalling (*Hgf*, *Col27a1* and *Lamc3*), degradation of the ECM (*Adamts5*), DNA damage/telomere stress‐induced senescence (*Hist1h4i*), cell cycle (*Mcm2*) and ECM organization (*Col12a1*) (Data S1). The results of the analyses confirmed the significantly decreased expression levels of *Hgf*, *Col27a1*, *Lamc3, Adamts5* and *Hist1h4i* (all *p* < .005; Figure 3G) and significantly increased expression levels of *Col12a1* and *Mcm2* (all *p* < .005; Figure 3G), consistent with the transcriptomics screening. Linear regression analysis showed that the Pearson coefficient between mRNA expression of the eight candidate genes detected by qPCR and RNA‐seq in the kidney tissues of six whole transcriptome sequenced animals was all higher than .8, indicating a good correlation between techniques (Figure S3G). Altogether, these findings demonstrate for the first time that *Men1* deficiency induces extensive changes in the transcriptional profile during fibrotic progression in the murine kidney.

### 3.4 Deletion of *Men1* induces tubular epithelial‐to‐mesenchymal transition

**FIGURE 4:**
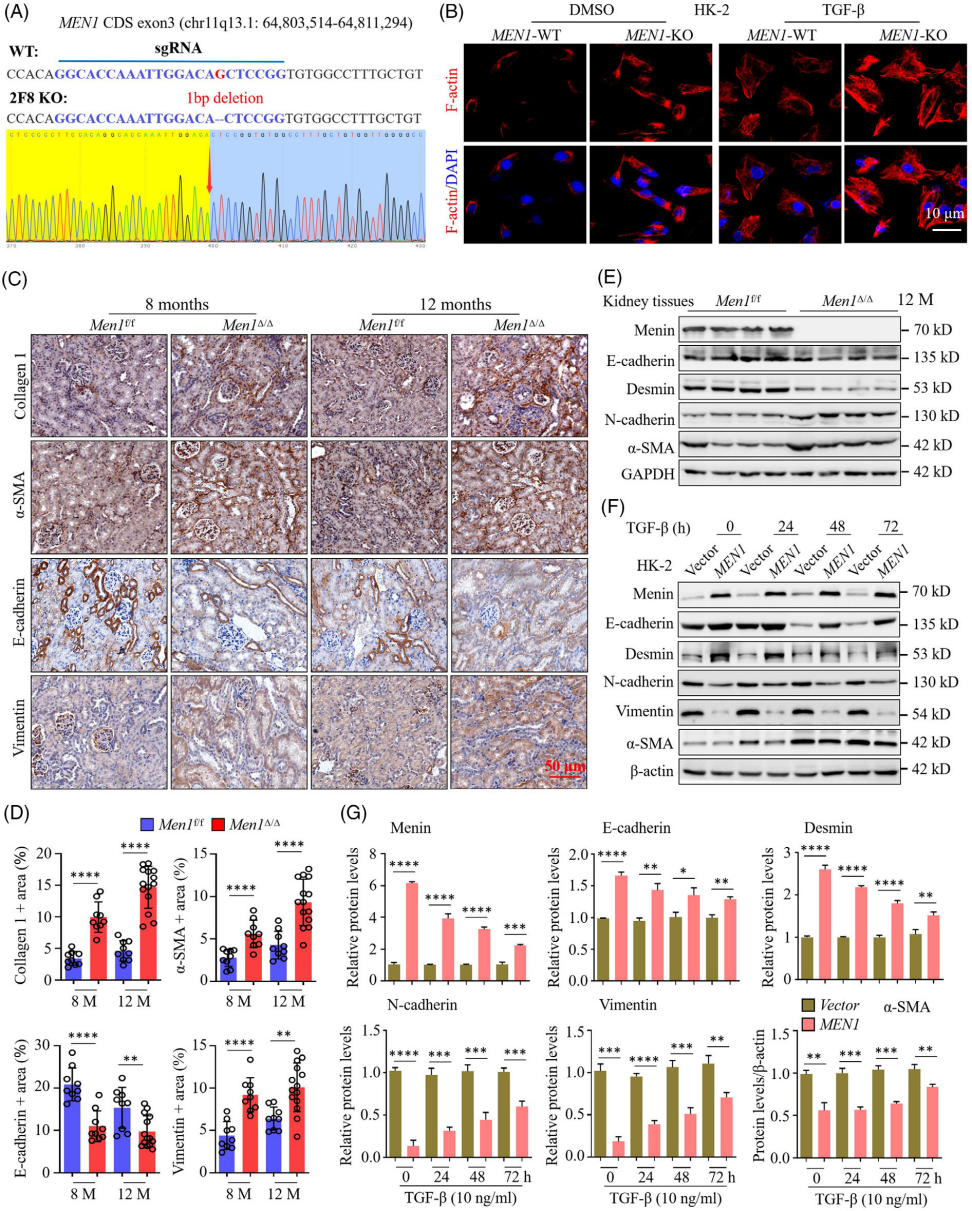
Deletion of Men1 induces tubular epithelial‐to‐mesenchymal transition. (A) Scheme of CRISPR/Cas9‐mediated knockout of MEN1 in HK‐2 cells and sgRNA sequence targeting MEN1 CDS exon3. (B) Immunofluorescence staining for F‐actin (red) and 4′, 6′‐diamidino 2‐phenylindole (DAPI, blue) in the MEN1‐WT and MEN1‐KO HK‐2 cells treated with 10‐ng/ml TGF‐β for 48 h; scale bars 10 μm. (C) Immunohistochemistry (IHC) staining for the indicated proteins in the kidney tissues of the *Men1* ^f/f^ and Men1^Δ/Δ^ mice; scale bars 50 μm. (D) Automatic quantification of collagen 1, α‐SMA, E‐cadherin and vimentin IHC staining in kidney tissues of the *Men1* ^f/f^ and Men1^Δ/Δ^ mice (*n* = 9 mice per group at 8 months, *n* = 9 mice in the *Men1* ^f/f^ and *n* = 14 mice in the Men1^Δ/Δ^ groups at 12 months). (E) Western blotting was used to detect the expression of the indicated proteins in the kidney tissues of the *Men1* ^f/f^ and Men1^Δ/Δ^ mice. (F) Western blotting was used to detect the expression of the indicated proteins in the vector‐ and MEN1‐HK‐2 cells after exposure to 10‐ng/ml TGF‐β. (G) Quantification of the greyscale image of indicated proteins in (F) (three biological replicates). The data are represented as mean ± standard deviation (SD);*p < .05, **p < .01, ***p < .001, ****p < .0001.

Numerous signalling pathways that are significantly enriched in the *Men1* ^Δ/Δ^ mouse kidney tissues, such as JAK‐STAT, PI3k‐Akt and G2/M checkpoints, have been reported as regulators of EMT.^23^, ^41^, ^42^ We speculate that *Men1* deletion leads to renal fibrosis in an EMT‐dependent manner and established CRISPR‐mediated *MEN1* KO HK‐2 cells (Figures 4A and S4A). We observed that *MEN1* KO induced the morphologic transformation in tubular epithelial cells, presenting a mesenchymal‐like cell phenotype (Figure S4B). HK‐2 cells displayed the classic cobblestone morphology of epithelial cells when grown in culture, whereas *MEN1*‐KO HK‐2 cells becoming elongated in shape, separating from adjacent cells and losing their cobblestone morphology; these effects were further exacerbated by treatment with TGF‐β (Figure S4B). IF staining showed that KO of *MEN1* notably induced F‐actin production, producing plentiful long stress fibres in HK‐2 cells with or without TGF‐β (Figure 4B). The results showed that menin is a key regulator that preserves functional fibroblastic morphology.

Furthermore, IHC staining showed that α‐SMA and collagen 1 staining was distinctly enhanced in kidney tissues of the *Men1* ^Δ/Δ^ mice at 8 and 12 months, confirming the fibrotic features of the kidney upon *Men1* deficiency (Figure 4C,D). We found that staining of E‐cadherin, an epithelial marker, was significantly reduced, whereas vimentin, a marker for fibroblast activation, was highly stained in the renal tubular area of the *Men1* ^Δ/Δ^ mice compared with the *Men1* ^f/f^ mice (Figure 4C,D). Consistently, the expression of E‐cadherin and desmin, another epithelial marker was notably decreased in kidney tissues of the *Men1* ^Δ/Δ^ mice, whereas α‐SMA and N‐cadherin were markedly augmented (Figures 4E and S4C). In contrast, menin overexpression enhanced the expression of epithelial markers in HK‐2 cells with or without TGF‐β treatment, whereas the levels of mesenchymal markers and α‐SMA were obviously decreased in a time‐dependent manner (Figure 4F,G). Similar results were obtained in *MEN1* overexpression HK‐2 cells treated with IL‐1β (Figure S4D,F). In addition, KO of *MEN1* substantially reduced the expression of E‐cadherin and desmin, and enhanced N‐cadherin, vimentin and α‐SMA, and cyclin D1 expression in the HK‐2 cells and mRTECs treated with TGF‐β or IL‐1β (Figure S4E,G–I). Similarly, strikingly decreased E‐cadherin and H3K4me3 levels and increased N‐cadherin, vimentin and α‐SMA expression were observed in the sh*MEN1* HK‐2 cells treated with TGF‐β (Figure S4J,K). Taken together, our data clearly indicate that *MEN1* deficiency activates fibroblasts and induces EMT in vivo and in vitro.

### 3.5 Menin‐dependent chromatin H3K4me3 modification is involved in regulating EMT

**FIGURE 5:**
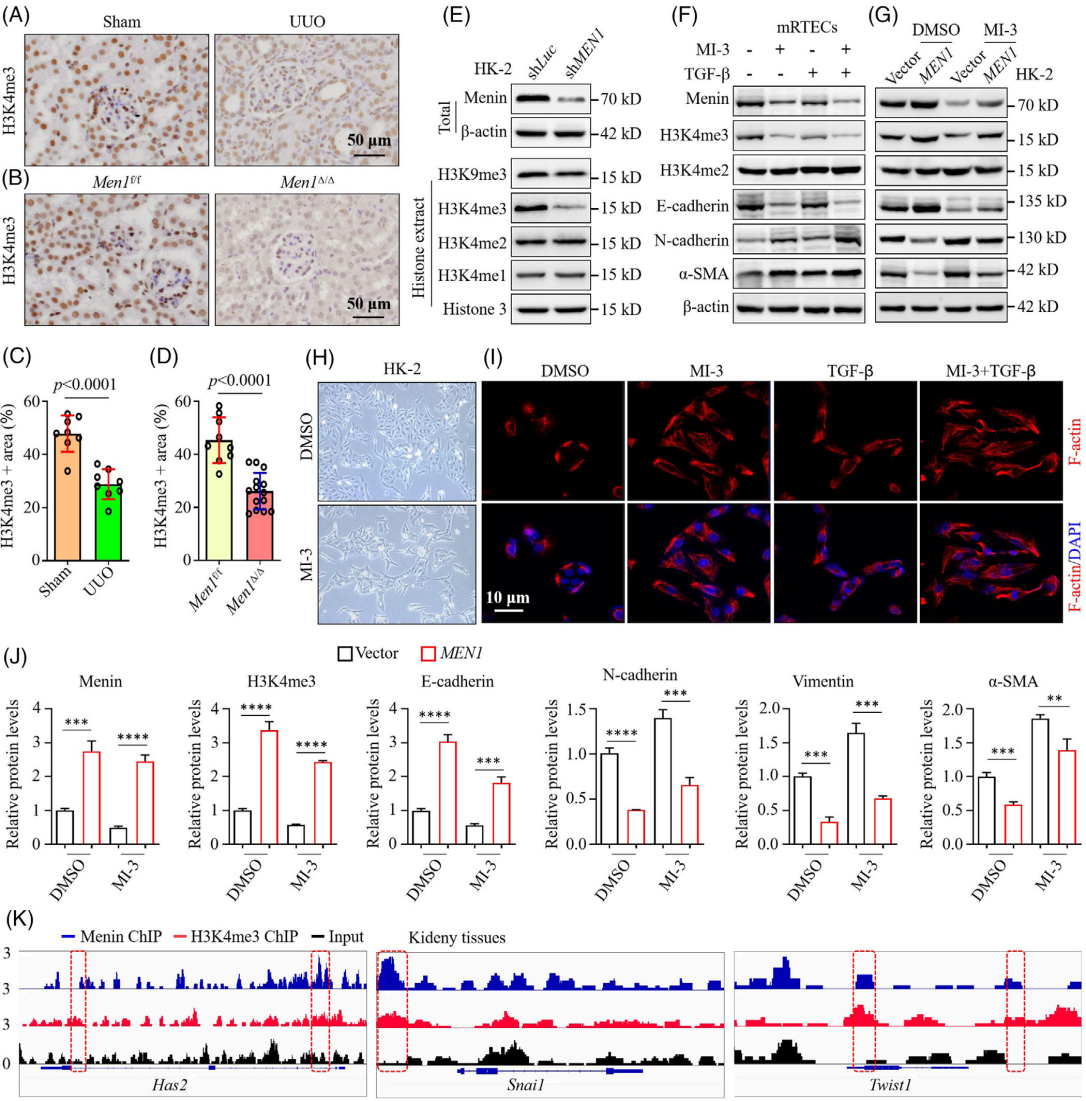
Menin‐dependent chromatin H3K4me3 modification is involved in regulating epithelial‐to‐mesenchymal transition (EMT). (A) Immunohistochemistry (IHC) staining for H3K4me3 in the kidney tissues of the sham and unilateral ureteral obstruction (UUO) mice 7 days after surgery (*n* = 8 mice per group); scale bars 50 μm. (B) IHC staining for H3K4me3 in the kidney tissues of the *Men1* ^f/f^ and Men1^Δ/Δ^ mice at 12 months; scale bars 50 μm. (C and D) Quantification of H3K4me3 IHC staining in (A) and (B), respectively. (E) Western blotting was used to detect the expression of menin in total lysates and modification of the indicated histones in histone extracts in the shLuc‐ and shMEN1‐HK‐2 cells. (F) Mouse renal tubular epithelial cells (mRTECs) were treated with 10‐μM MI‐3 for 2 days before TGF‐β treatment and were collected 2 days after exposure to 10‐ng/ml TGF‐β, and Western blotting was used to detect the expression of the indicated proteins. (G) Western blotting was used to detect the expression of the indicated proteins in the vector‐ and MEN1‐HK‐2 cells 72 h after 10‐μM MI‐3 treatment. (H) Representative morphological image of HK‐2 cells treated with 10‐μM MI‐3 for 72 h; original magnification ×100. (I) Immunofluorescence (IF) staining for F‐actin (red) and DAPI (blue) in the HK‐2 cells treated with 10‐μM MI‐3 for 72 h; scale bars 10 μm. (J) Quantification of the greyscale image of the indicated proteins in (G) (three biological replicates). (K) Integrated genomics view of menin and H3K4me3 chromatin binding at the Has2, Snail and Twist1 gene loci in the kidney tissues of the *Men1* ^f/f^ and Men1^Δ/Δ^ mice at 12 months. The data are represented as the mean ± standard deviation (SD); *p < .05, **p < .01, ***p < .001, ****p < .0001.

Chromatin histone remodelling is implicated in the development and progression of renal fibrosis through regulating the EMT process induced by TGF‐β signalling.^43^ Here, we found that the UUO mouse kidney tissues exhibited weak H3K4me3 staining relative to sham mice (Figure 5A,C). Exposure to TGF‐β or AA resulted in a time‐dependent decrease in H3K4me3 but not H3K4me2 or H3K4me1 in NRK‐52E cells or mRTECs (Figures 1G,K and S1L,M). Further IF staining analysis confirmed that low‐dose (2 ng/ml) TGF‐β treatment of HK‐2 cells caused a prominent reduction in nuclear H3K4me3 staining in a time‐dependent fashion (Figure S5A,B). These results suggest that low levels of H3K4me3 modification are an important indicator of kidney fibrogenesis. Given the well‐known effect of menin/MLL complex‐mediated H3K4me3 modifications on cell proliferation, apoptosis and tumourigenesis, we attempted to investigate whether menin excision‐induced EMT and fibrogenesis are associated with aberrant H3K4me3 modification. IHC revealed that fibrotic renal tissues of the *Men1* ^Δ/Δ^ mice clearly decreased H3K4me3 and H3K9me3 staining compared with that of the *Men1* ^f/f^ mice, whereas there was no obvious change in H3K4me2 staining (Figures 5B,D and S5C,D). Cellular histone analysis showed that sh*MEN1* notably decreased the modifications of chromatin H3K4me3 and H3K9me3 but not the modifications of H3K4me2 or H3K4me1 in HK‐2 cells (Figure 5E). Similar results were also found in the *MEN1*‐KO HK‐2 cells (Figure S5E). These data indicate that menin/MLL1‐mediated chromatin H3K4me3 modification is required for preventing kidney fibrogenesis.

As expected, MI‐3 (a specific inhibitor of menin/MLL1 interaction) strikingly attenuated the expression of menin, H3K4me3 (but not H3K4me2) and E‐cadherin but promoted N‐cadherin and ɑ‐SMA, and these effects were intensified by treatment with TGF‐β (Figures 5F and S5F). Exposure to MI‐3 resulted in greatly morphologic changes, with cells becoming elongated in shape (Figure 5H). IF staining showed that MI‐3 notably induced F‐actin production, resulting in a mass of long stress fibres in HK‐2 cells (Figure 5I). Overexpression of *MEN1* evidently enhanced the expression of H3K4me3 and E‐cadherin and suppressed the expression of N‐cadherin and α‐SMA in HK‐2 cells; intriguingly, these effects were neutralized by MI‐3 incubation (Figure 5G,J). Importantly, sh*MEN1*‐induced decreases in H3K4me3 and E‐cadherin and the enhancement of N‐cadherin and α‐SMA in HK‐2 cells were effectively reversed by SP2509, an inhibitor specifically targeting LSD1, which is responsible for methylating H3K4me3^44^ (Figure S5G,H). These results indicate that the remodelling of H3K4me3 effectively restores *MEN1* dysfunction‐actuated fibrogenic EMTs.

We next determined the epigenomic effect of menin‐mediated chromatin H3K4me3 on EMT and performed ChIP‐seq for menin and H3K4me3 in 12‐month‐old *Men1* ^f/f^ mouse kidney tissues using either Hi‐Seq or NextSeq platforms with inputs as controls (Data S2). We found that menin and H3K4me3 were extensively enriched in some EMT gene bodies and the genome binding pattern of menin overlapped with that of H3K4me3, including in the EMT drivers *Has2*, *Snai1*, *Twist1* and *Zeb2* (Figure 5K and Data S2) but not in *Smad3* or *Smad6* (data not shown). These data support the conclusion that menin/MLL1 mediated chromatin H3K4me3 modification to prevent EMT and renal fibrogenesis.

### 3.6 Hgf‐Adamts5 is a novel target gene that is epigenetically regulated by menin

**FIGURE 6:**
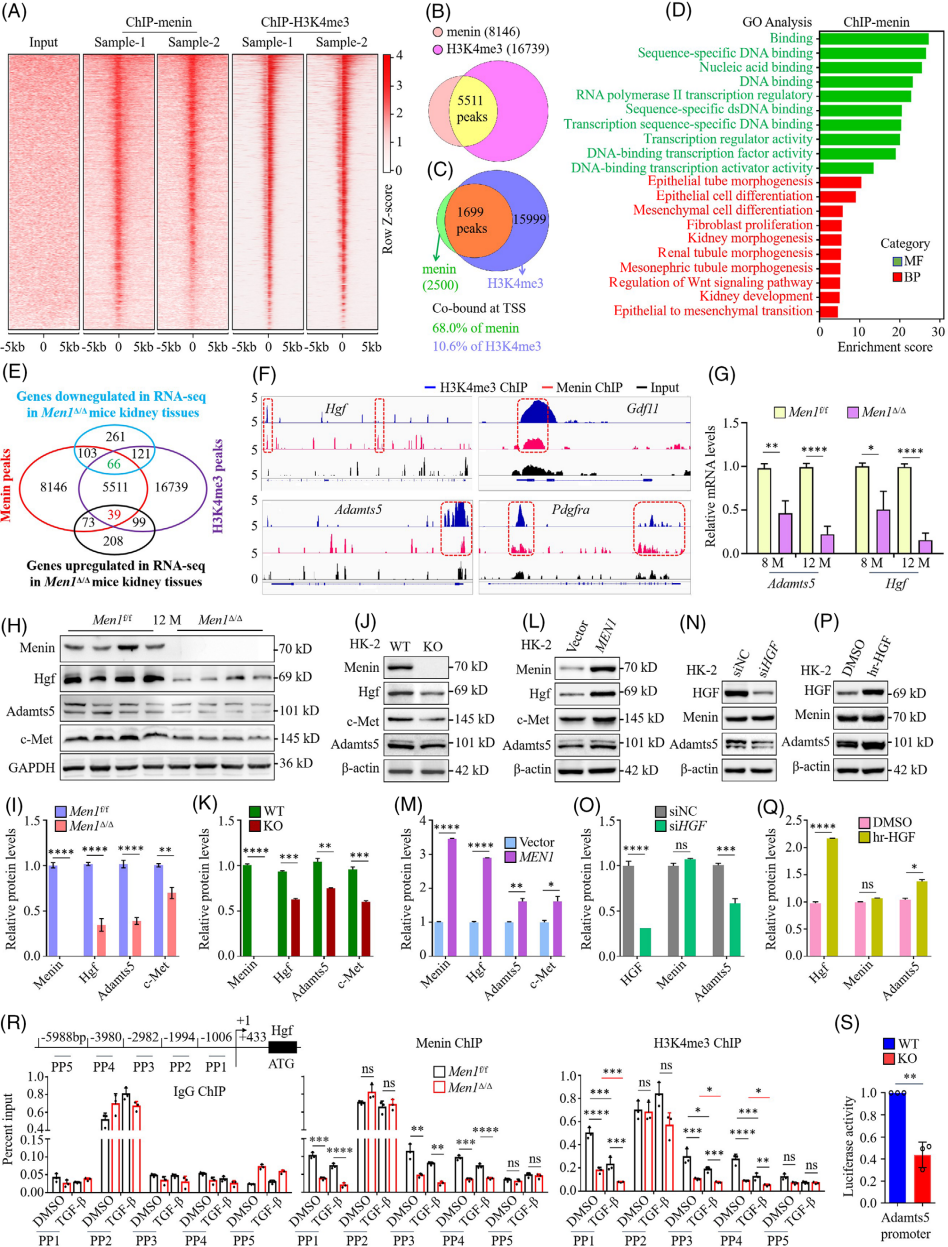
Hgf/Adamts5 is a novel target gene that is epigenetically regulated by menin. (A) Heat maps of read distribution of menin and H3K4me3 peaks within 5 kb of the transcription start sites in the kidney tissues of the *Men1* ^f/f^ mice at 12 months. (B) Conserved binding peaks were respectively defined between two biological replicates of menin and H3K4me3 based on chromatin immunoprecipitation‐sequencing (ChIP‐seq) data, and a Venn diagram shows the overlap of conserved binding peaks of menin and H3K4me3. (C) Venn diagram showing the overlap of genes occupied by menin and enriched for H3K4me3 at the TSSs. (D) Gene ontology (GO) enrichment analysis showing representative biological processes (BPs) and molecular functions (MFs) for significantly enriched fibrosis‐related genes based on the menin ChIP‐seq data. (E) Venn diagram showing the overlap among the genes annotated to menin and H3K4me3 binding peaks and genes with up‐ or downregulated expression in the RNA‐sequencing (RNA‐seq). (F) Integrated genomics view of menin and H3K4me3 chromatin binding at the loci of the targeted genes in kidney tissues of the *Men1* ^f/f^ mice at 12 months. (G) Quantitative real‐time PCR (qPCR) was used to detect the mRNA expression of Hgf and Adamts5 in the kidney tissues of the *Men1* ^f/f^ and Men1^Δ/Δ^ mice. (H) Western blotting was used to detect the expression of the indicated proteins in the kidney tissues of the *Men1* ^f/f^ and Men1^Δ/Δ^ mice (*n* = 4 mice per group). (I) Quantification of the greyscale image of the indicated proteins in (H). (J) Western blotting was used to detect the expression of the indicated proteins in the MEN1‐WT and MEN1‐KO HK‐2 cells. (K) Quantification of the greyscale image of the indicated proteins in (J) (three biological replicates). (L) Western blotting was used to detect the expression of the indicated proteins in the vector and MEN1‐HK‐2 cells. (M) Quantification of the greyscale image of the indicated proteins in (L) (three biological replicates). (N) Western blotting was used to detect the expression of the indicated proteins in HK2 cells transfected with siRNA for 48 h. (O) Quantification of the greyscale image of the indicated proteins in (N) (three biological replicates). (P) Western blotting was used to detect the expression of the indicated proteins in HK‐2 cells treated with 10‐ng/ml rh‐HGF for 72 h. (Q) Quantification of the greyscale image of the indicated proteins in (P) (three biological replicates). (R) Schematic representation of the Hgf gene promoter regions and primer pairs (PP) used for ChIP assays. ChIP‐qPCR was performed with anti‐menin or anti‐H3K4me3 antibodies on samples from the immortalized *Men1* ^f/f^ and Men1^Δ/Δ^ RTECs and IgG served as the negative control (three biological replicates). (S) Impact of MEN1 knockout on Adamts5 promoter activity in HK‐2 cells by luciferase reporter analyses. The data are represented as the mean ± standard deviation (SD); *p < .05, **p < .01, ***p < .001, ****p < .0001.

Next, we sought to understand the mechanistic basis of menin's regulation of EMT during renal fibrogenesis. We analysed menin and H3K4me3 ChIP‐seq data in two mouse kidney tissues (Figures 6A and S6A). The heat maps show the peak enrichment levels at transcription start sites (TSSs) in a ±5‐kb window, and menin and H3K4me3 ChIP‐seq in the two biological replicates displayed highly similar genome distributions, confirming the close reproducibility between both ChIP‐seq replicates (Figure 6A). We observed wide and dispersive menin peaks around the TSSs, indicating that the signal is spread across the TSSs, whereas narrow and sharp H3K4me3 peaks around the TSS indicate high levels of H3K4me3 binding (Figures 6A and S6A). Peak detection data defined (−10log (*p* value) ≥ 30, fold enrichment ≥ 2.5) 8146 conserved menin‐enriched regions, composed of 12.9% promoter, 33.9% intergenic, 33.3% intron, 2.0% exon and 17.8% upstream regions, and 16 739 conserved H3K4me3‐enriched regions were identified with H3K4me3 bound at 93.5% of the gene promoters (Figure S6B). A Venn diagram demonstrates a significant geneome‐wide colocalization (*n* = 5511) of the peak binding sites for menin and H3K4me3 enrichment (Figure 6B). Importantly, the vast majority (68.0%) of the menin‐occupied TSS regions were marked with prominent H3K4me3 modifications, whereas menin was merely bound at 10.6% of the H3K4me3‐enriched regions (Figure 6C), suggesting that menin regulates gene expression greatly dependent on chromatin H3K4me3 modification. Furthermore, the analysis of GO BPs indicated that menin‐enriched genes are involved in processes related to fibrogenesis, including EMT, regulation of Wnt signalling pathway, renal tubule morphogenesis, fibroblast proliferation and epithelial cell differentiation (false discovery rate (FDR) < .001, Figure 6D); the pattern of H3K4me3 binding to genes was very similar to that of menin, with a clear enrichment in the Wnt signalling pathway, epithelial cell proliferation/differentiation and G2/M phase transition in BP analysis (FDR < 1.87e−13, Figure S6C), whereas MFs mainly included DNA/RNA binding, transferase activity, transcription factor binding and transcription regulatory region sequence‐specific DNA binding (FDR < 1.62e−39, Figure S6D). These data indicate that menin and H3K4me3 are recruited to key genes controlling EMT and fibrosis‐related pathways in renal fibrogenesis.

Menin regulates gene‐expression‐dependent MLL1‐mediated histone methyltransferase activity in the promoter region. We suspect that menin regulates EMT and fibrosis by affecting H3K4me3 distribution. A Venn diagram showing genes that are bound by menin and H3K4me3 specifically in kidney tissues and 261 genes had downregulated expression and 208 genes had upregulated expression in the absence of *Men1* (Figure 6E). We found a set of 66 genes with downregulated expression and a set of 39 genes with upregulated expression and also cobound to menin and H3K4me3 upon *Men1* deficiency (Figure 6E). Several genes with upregulated expression, such as *Wnt5b* and *Bmp6*, have been reported to be implicated in Wnt pathway activation and hepatic fibrosis^34^, ^45^ (Figure S6E and Data S2). Of special interest in this set of genes with downregulated expression, including *Hgf*, *Adamts5*, *Gdf11* and *Pdgfra*, which are participated into the myofibroblast activation, ECM deposition and renal fibrogenesis^29^, ^30^, ^46^ (Figure S6E and Data S2). According to our ChIP‐seq data, these genes demonstrated a significant enrichment of menin and H3K4me3 binding to the promoter or upstream region (Figures 6F and S6F), and then we focused on two potential candidates, *Hgf* and *Adamts5*. Our data revealed that the protein expression of Adamts5 were markedly diminished in kidney tissues of the UUO mice when compared with the sham mice (Figure S6G,I); exposure to TGF‐β resulted in a time‐dependent reduction in *Hgf* and *Adamts5* mRNA expression in mRTECs and HK‐2 cells (Figure S6K,L). Importantly, loss of *MEN1* memorably abrogated the mRNA and protein expression of Hgf, c‐Met (an Hgf receptor) and Adamts5 in kidney tissues and HK‐2 cells (Figures 6G–K and S6L). Conversely, the overexpression of menin clearly promoted the levels of Hgf, c‐Met and Adamts5 protein in HK‐2 cells (Figure 6L,M). Interestingly, the knockdown (KD) of HGF (si*HGF*) prominent diminished the expression of Adamts5 protein (Figure 6N,O), whereas exogenous recombinant human HGF (rh‐HGF) obvious elevated the expression of Adamts5 (Figure 6P,Q), but the levels of menin were not clearly altered by ectopic expression of HGF (Figure 6N–Q). These findings indicate that Hgf/Adamts5 are novel downstream target genes regulated by menin.

Next, we investigated the molecular mechanism by which menin upregulates Hgf/Adamts5 expression. ChIP‐qPCR results show that menin specifically binds to the promoter regions of the *Hgf* and *Adamts5* genes, and KO of *Men1* observably reduces menin binding and H3K4me3 levels in immortalized mRTECs (Figures 6R and S6M). Notably, exposure to TGF‐β led to a marked reduction in the enrichment of menin and H3K4me3 at the promoter regions of these target genes (Figures 6R and S6M). Finally, a luciferase reporter assay showed that KO of *MEN1* distinctly decreased *Adamts5* promoter activity (Figure 6S). Consistently, *Adamts5* promoter activity was dramatically enhanced by overexpression of menin in HK‐2 cells (Figure S6N). Altogether, these findings show that menin promotes transcriptional activation of *Hgf* and *Adamts5* through an H3K4me3‐dependent epigenetic mechanism.

### 3.7 Recombinant human HGF (rh‐HGF) ameliorates renal fibrosis induced by *Men1* deletion

**FIGURE 7:**
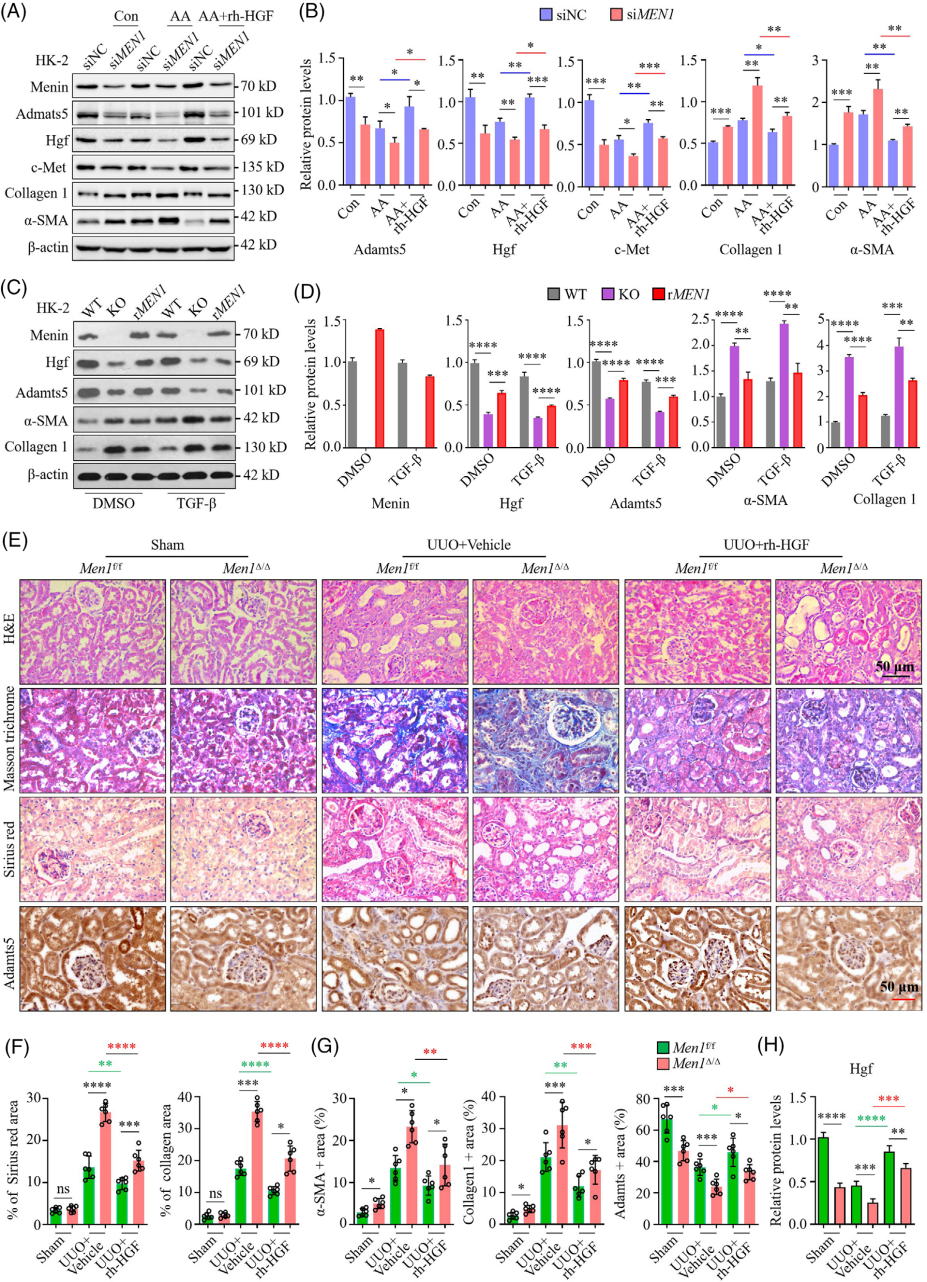
Recombinant human hepatocyte growth factor (HGF) ameliorates renal fibrosis induced by Men1 deletion. (A) HK‐2 cells were transfected with siRNA for 48 h and then treated with 5‐μg/ml aristolochic acid (AA) alone or combined 10‐ng/ml rh‐HGF for 48 h. Western blotting was used to detect the expression of the indicated proteins in the siNC‐ and siMEN1‐transfected HK‐2 cells. (B) Quantification of the greyscale image of the indicated proteins in (A) (three biological replicates). (C) HK‐2 cells with wild‐type MEN1 (WT), depleted MEN1 (KO) and reconstituted MEN1 (rMEN1) expression were treated with 10‐ng/ml TGF‐β for 72 h. Western blotting was used to detect the expression of the indicated proteins. (D) Quantification of the greyscale image of the indicated proteins in (C) (three biological replicates). (E) Representative haematoxylin–eosin (H&E), Masson's trichome and Sirius red staining images, and immunohistochemistry (IHC) staining for Adamts5 in kidney sections from the vehicle‐ and rh‐HGF‐treated *Men1* ^f/f^ and Men1^Δ/Δ^ mice; scale bars 50 μm. (F) Quantification of the area of Masson's trichrome and Sirius red staining in (E). (G) Quantification of α‐SMA, Collagen 1, and Adamts5 IHC staining in (E) and Figure S7H. (H) Quantification of the greyscale image of Hgf proteins in Figure S7I (*n* = 4 per group). The data are represented as mean ± standard deviation (SD); *p < .05, **p < .01, ***p < .001, ****p < .0001.

Endogenous HGF signalling is essential to protect the RTECs phenotype by interdicting epithelial‐to‐myofibroblastic conversion.^29^ Here, we observed that exogenous rh‐HGF exposure prominently promoted the expression of Adamts5, Hgf and c‐Met whereas prevented α‐SMA expression but did not clearly affect the protein level of menin in primary mRTECs (Figure S7A,B). Importantly, rh‐HGF partly reversed the inhibition of Adamts5 and c‐Met expression induced by MI‐3 treatment and attenuated the elevation of α‐SMA expression reaching statistical significance (Figure S7A,B). In addition, AA exposure predominantly decreased the expression of Adamts5, Hgf and c‐Met and increased the expression of collagen 1 and α‐SMA, and this effect was further enhanced by si*MEN1* (Figure 7A,B). As expected, rh‐HGF partially rescued the suppression of Adamts5 and c‐Met expression induced by si*MEN1* treatment while counteracting the promotion of collagen 1 and α‐SMA expression (Figure 7A,B). Consistently, the depletion of *MEN1* largely dampened the expression of Hgf and Adamts5, enhanced α‐SMA and collagen 1 expression, and this effect induced by *MEN1* KO was partly rescued by reconstituted expression of wild type *MEN1* (r*MEN1*) in HK‐2 cells with or without TGF‐β (Figure 7C,D).

We further attempted to evaluate the pharmacological effects of rh‐HGF on renal fibrosis in vivo. We administered rh‐HGF by *i.v*. into the *Men1* ^f/f^ and *Men1* ^Δ/Δ^ mice with TIF induced by UUO (Figure S7C). The mice were sacrificed at Day 3 post–rh‐HGF treatment, in which there were no significant changes in kidney weight, blood glucose and serum creatinine in the sham and UUO *Men1* ^f/f^ and *Men1* ^Δ/Δ^ mice (Figure S7D–F). At the time of sacrifice, however, the *Men1* ^Δ/Δ^ mice in the UUO but not in the sham‐operated mice had relatively severer interstitial fibrosis when compared to those of the *Men1* ^f/f^ mice (Figure S7G). UUO resulted in a substantial enhance in collagen deposition in obstructed kidneys of both the *Men1* ^f/f^ and *Men1* ^Δ/Δ^ mice, whereas loss of *Men1* further increased collagen accumulation, showed as a greater Sirius red and Masson's trichrome staining area (Figure 7E,F). IHC and immunoblotting for the detection of collagen 1 and α‐SMA proteins expression in kidney tissues confirmed the results obtained after Masson's trichrome and Sirius red staining (Figures 7G and S7H–J). Of note, UUO led to a significant diminish in the expression of Hgf, c‐Met and Adamts5, and these effects were further aggravated by *Men1* deficiency (Figures 7E,G,H and S7I,J). Strikingly, exogenous HGF administration significantly ameliorated the *Men1* deficiency‐induced degree of kidney injury and the severity of renal fibrosis in the mice that underwent UUO compared with that in the vehicle group, as determined by H&E, IHC and Western blot analysis (Figures 7E–H and S7G–J). Consistent with the in vitro results, rh‐HGF treatment notably restored the expression of Hgf, c‐Met and Adamts5, whereas reduced the collagen 1 and α‐SMA staining in kidney tissues of the *Men1* ^Δ/Δ^ mice compared with the vehicle‐treated mice (Figures 7E,G,H and S7H–J). The fibrotic/collagen‐positive areas were not significantly different between the *Men1* ^f/f^ and *Men1* ^Δ/Δ^ mice in the sham‐operated kidneys (Figure 7E,F). Altogether, these results further support the renoprotective roles of the *MEN1* gene in the pathogenesis of kidney fibrosis and demonstrate that pharmacological intervention with rh‐HGF significantly ameliorated *Men1* deletion‐exacerbated renal fibrosis.

### 3.8 Deletion of *MEN1* results in G2/M arrest and JNK signalling pathway activation

**FIGURE 8:**
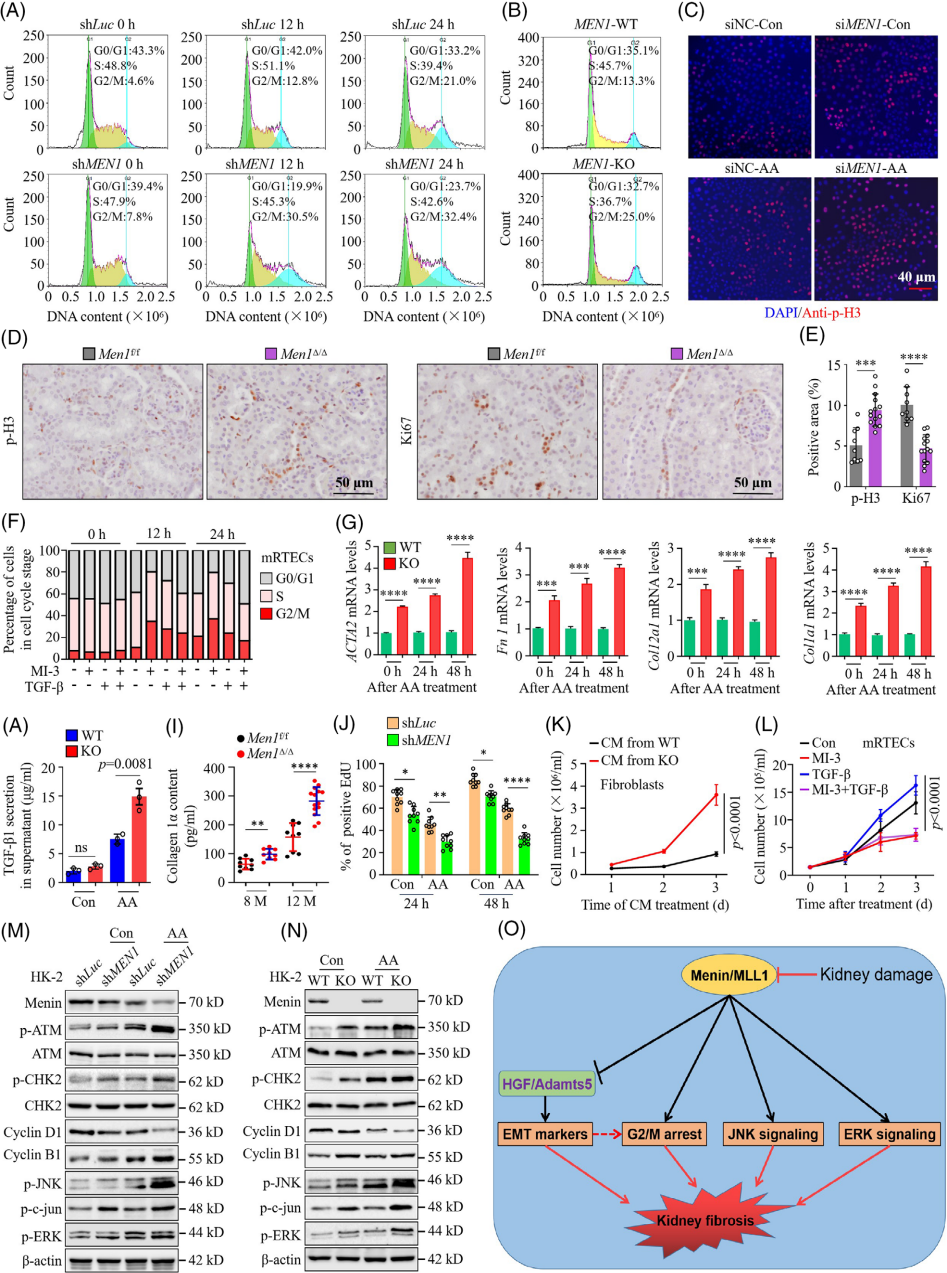
Deletion of MEN1 results in G2/M arrest and JNK signalling pathway activation. (A) Cell cycle analysis by propidium iodide (PI) staining and flow cytometry in the shLuc‐ and shMEN1‐HK‐2 cells after 5‐μg/ml aristolochic acid (AA) treatment. (B) Cell cycle analysis by PI staining in the MEN1‐WT and MEN1‐KO cells treated with 5‐μg/ml AA for 24 h. (C) Immunofluorescence (IF) staining for phosphorylated histone H3 (p‐H3) (red) and DAPI (blue) in the siNC‐ and siMEN1‐HK‐2 cells treated with 5‐μg/ml AA for 24 h; scale bars 40 μm. (D) Immunohistochemistry (IHC) staining for p‐H3 and Ki67 in the kidney tissues of the *Men1* ^f/f^ and Men1^Δ/Δ^ mice at 12 months; scale bars 50 μm. (E) Quantification of p‐H3 and Ki67 IHC staining in (D) (*n* = 9 mice per group at 8 months, *n* = 9 mice in the *Men1* ^f/f^ and *n* = 14 mice in the Men1^Δ/Δ^ groups). (F) Cell cycle distribution in primary mouse renal tubular epithelial cells (mRTECs) at the indicated time points after 10‐μM MI‐3 or 10‐ng/ml TGF‐β alone or combined treatment. (G) Quantitative real‐time PCR (qPCR) was used to detect the mRNA expression of the indicated genes in the MEN1‐WT and MEN1‐KO HK‐2 cells after 5‐μg/ml AA treatment. (H) Enzyme‐linked immunosorbent assays (ELISAs) were used to measure the 10‐ng/ml TGF‐β1 concentration in the supernatant of the MEN1‐WT and MEN1‐KO HK‐2 cells treated with 5‐μg/ml AA for 48 h. (I) ELISA were used to measure the content of collagen 1α in the serum of the *Men1* ^f/f^ and Men1^Δ/Δ^ mice (*n* = 9 mice per group at 8 months, *n* = 9 mice in the *Men1* ^f/f^ and *n* = 14 mice in the Men1^Δ/Δ^ groups at 12 months). (J) Quantification of EdU‐positive cells in Figure S8H (*n* = 10 images per group). (K) Proliferation of fibroblasts incubated with conditioned medium (CM) from the MEN1‐WT and MEN1‐KO HK‐2 cells treated with 5‐μg/ml AA (three biological replicates). (L) Proliferation of primary mRTECs was determined by using trypan blue exclusion assay after 10‐μM MI‐3 or 10‐ng/ml TGF‐β alone or combined treatment (three biological replicates); the data are represented as mean ± standard deviation (SD) (one‐way ANOVA). (M) Western blotting was used to detect the expression of the indicated proteins in the shLuc‐ and shMEN1‐HK‐2 cells treated with 5‐μg/ml AA for 48 h. (N) Western blotting was used to detect the expression of the indicated proteins in the MEN1‐WT and MEN1‐KO HK‐2 cells treated with 5‐μg/ml AA for 48 h. (O) A schematic that summarizes the flow of signals from initial kidney damage to the suppression of menin/MLL1 expression, the expression of hepatocyte growth factor (HGF)‐Adamts5 and the activation of multiple signalling pathways involved in kidney fibrosis. The data are represented as mean ± SD; *p < .05, **p < .01, ***p < .001, ****p < .0001.

We recently found that deletion of *MEN1* activated phosphorylation of CHK2 (p‐CHK2), a G2/M cell cycle checkpoint, in lung cancer cells.^25^ Considering that cell cycle G2/M arrest in RTECs gives rise to renal fibrosis,^23^ we further investigated whether menin‐initiated renal fibrosis is associated with G2/M arrest in RTECs. Indeed, the percentage of sh*Luc* cells in G2/M phase increased from 13% at 12 h to approximately 20% at 24 h in HK‐2 cells treated with AA; however, the percentage of sh*MEN1* cells in G2/M phase increased to >30% 12‐h post‐AA treatment and maintained at a steady level during the observation period (30.5% ± 2.6% at 24 h) (Figures 8A and S8A). Similar results were obtained when analysing the *MEN1‐*KO in HK‐2 cells exposed to AA for 24 h compared with that of the *MEN1*‐WT HK‐2 cells (Figure 8B). Moreover, *MEN1‐*KO prominently enhanced the cell number in G2/M phase in HK‐2 cells, as confirmed by the increased IF staining for phosphorylated histone 3 at Ser10 (p‐H3), a typical marker for the cell cycle G2/M phase,^47^ that staining was intensified by AA treatment (Figures 8C and S8B). Similarly, the deletion of *Men1* signally increased the p‐H3 positive staining and reduced Ki67 staining in kidney tissues relative to the 12‐month *Men1* ^f/f^ mice (Figure 8D,E). These results are consistent with observations in mRTECs treated with MI‐3, which resulted in G2/M arrest in a time‐ and dose‐dependent fashion (Figures 8F and S8C,D). Treatment with MI‐3 obviously postponed cell cycle progression and arrested cells in G2/M phase, which was accelerated by TGF‐β to reach levels like those of the control cells (Figures 8F and S8C). These data indicate that ectopic expression of *MEN1* elongated the cell cycle G2/M phase and amassed G2/M cell populations in human or mouse RTECs.

Next, we investigated whether menin‐mediated G2/M arrest of RTECs is involved in renal fibrogenesis. We found that treatment of HK‐2 cells with paclitaxel (TAX), a G2/M arrest inducer,^48^ for 24 h triggered significant cell cycle G2/M arrest (Figure S8E), with an upregulation of the mRNA expression of *ACTA2*, *Fn1* and *Col12a1* in a dose‐dependent manner (Figure S8F). Similarly, treatment with AA led to a marked elevation in the mRNA expression of *ACTA2*, *Fn1*, *Col12a1* and *Col1a1*, and importantly, these effects were further aggravated by the KO of *MEN1* (Figure 8G). In addition, *MEN1*‐KO significantly promoted the protein secretion of TGF‐β1 and collagen 1α induced by AA in HK‐2 cells but had no effect on their basal levels (Figures 8H and S8G). We also observed an outstanding enhance in the content of serum collagen 1α in the 8‐ and 12‐ month fibrotic *Men1* ^Δ/Δ^ mice expressing high levels of profibrotic factors compared with the *Men1* ^f/f^ mice (Figure 8I). These results suggest that *Men1* deficiency‐induced kidney fibrogenesis is implicated in G2/M arrest of the cell cycle.

Cell cycle G2/M arrest of RTECs affected the proliferating efficiency of epithelial cells. Indeed, we showed that KD of *MEN1* resulted in time‐dependent inhibition of the proliferation of HK‐2 cells treated with or without AA, as confirmed by a decreased in number of EdU‐positive cells (Figures 8J and S8H). Similarly, after MI‐3 or TAX treatment, the growth inhibition of mRTECs obviously increased relative to that of the control (Con) group (Figure S8I). Interestingly, the CM form the AA‐treated *MEN1*‐KO HK‐2 cells conspicuously promoted the cell proliferation and collagen 1α production of fibroblasts compared with that of the *MEN1*‐WT HK‐2 cells (Figures 8K and S8J). We suspect that the observed phenotype was the consequence of profibrogenic growth factors secreted from the *MEN1*‐KO HK‐2 cells induced by AA treatment. As expected, exogenous TGF‐β resulted in an obvious increase in the proliferative efficiency in mRTECs, whereas these effects were substantially counteracted by treatment with MI‐3 (Figure 8L). Activation of the cell cycle checkpoint CHK2, which is the downstream of the ATM (ataxia telangiectasia mutated) pathway, induces cell cycle G2M arrest in proximal tubular epithelial cells.^49^ We found that KD of *MEN1* distinctly triggered the phosphorylation of CHK2 (p‐CHK2) and p‐ATM and augmented the expression of cyclin B1 whereas decreased the cyclin D1 expression in HK‐2 cells with or without AA (Figures 8M and S8K,M). We also observed that the KD of *MEN1* prominently stimulated the activation of JNK signalling induced by AA exposure, with an enhanced expression of p‐JNK and p‐c‐jun, which was consistent with periods when G2/M arrest was notable (Figures 8K and S8M). Moreover, the ERK was significantly activated in the sh*MEN1* cells relative to the sh*Luc* HK‐2 cells, as shown by enhanced activation of phosphorylated ERK (p‐ERK) (Figures 8K and S8M). Similar results were obtained by repeating this experiment in the *MEN1*‐WT and *MEN1*‐KO HK‐2 cells exposed to AA (Figures 8N and S8L,N).

Studies have reported that apoptosis in RTEC has been involved in the occurrence and development of renal fibrosis.^50^ Indeed, TGF‐β treatment resulted in a substantial increase in apoptosis of the sh*Luc‐* and sh*MEN1* HK‐2 cells (Figure S8O,P). However, there were no significant differences in the apoptosis rate or the mRNA expression of apoptosis‐associated genes, such as *Bax*, *Bcl2* and *Aparf*, in the sh*Luc* and sh*MEN1* HK‐2 cells with or without TGF‐β (Figure S8O,P, and data not shown). Similarly, the KO of *MEN1* induced G2/M arrest and promoted profibrotic genes but did not cause obviously increased apoptosis in HK‐2 cells with or without AA (Figure S8Q). These results support the conclusion that *Men1* deficiency‐induced G2/M arrest of cell cycle leads to renal fibrosis independent of the apoptosis pathway. Thus, the decreased epithelial cell numbers associated with renal fibrosis may be explained by G2/M arrest of the cell cycle rather than increased apoptosis.

## 4 DISCUSSION

In the present study, we describe the crucial role of *MEN1* in the control of renal fibrogenesis progression and establish the functional importance of *MEN1* expression in the kidney, showing that *MEN1* protects the kidney against fibrotic injury. We initially determined the expression of menin, MLL1 and H3K4 methylation in profibrogenic factor‐treated RTECs as well as in the kidneys of fibrotic mice or DN and diabetic patients; we presumed that the expression of the menin/MLL1 complex in kidney tubular injury was associated with renal fibrosis. As expected, the ablation of *MEN1* significantly induced ECM deposition, EMT signalling and chronic renal fibrosis in the *Men1* ^Δ/Δ^ mice compared with the *Men1* ^f/f^ mice. We propose an epigenetic activation mechanism by which menin/MLL1 regulates Hgf‐Adamts5 during renal fibrogenesis. Strikingly, exogenous rh‐HGF reduced ECM accumulation and blocked renal fibrosis in the obstructed kidneys of the *Men1* ^Δ/Δ^ mice after UUO. In addition, we also observed that *MEN1* deficiency significantly triggered G2/M arrest and concurrently activated JNK signalling. Together, these results suggest that menin expression could prevent the progression of renal fibrosis.

Kidney fibrosis is the ultimate manifestation of chronic kidney disease, in which TGF‐β is considered a pivotal mediator of fibrotic signalling in RTECs. TGF‐β‐induced tubular epithelial cells undergo EMT characterized by loss of epithelial characteristics and gain of mesenchymal markers.^51^ We noted that TGF‐β resulted in a dose‐ and time‐dependent reduction in the mRNA and protein expression of MEN1, indicating a potential role of this molecule in EMT of RTECs. Previous studies have demonstrated that menin interacts with β‐catenin, a pivotal mediator of the classical Wnt signalling pathway that regulates EMT activation and inhibits islet tumour cell proliferation.^52^ The results of our study show that *MEN1* deficiency caused an obvious reduction in the epithelial markers and an elevation of the expression of mesenchymal markers, which was consistent with our recent reports in lung adenocarcinoma.^25^ However, the overexpression of *MEN1* blunted the TGF‐β‐induced expression of vimentin and α‐SMA. KO of *MEN1* in HK‐2 cells triggered a morphological transition of tubular epithelial cells to a mesenchymal phenotype. Although myofibroblast activation is important for the initiation of kidney fibrosis, EMT has been shown to be a major determinant of fibrotic progression and irreversibility.^53^ Our data demonstrate that *MEN1* deficiency‐mediated high levels of EMT signalling are a momentous mechanism that may lead to the transformation of damaged renal tubular cells into mesenchymal cells.

*MEN1* tends to be involved in anti‐tumour^54^ and anti‐inflammatory^55^ effects and suppression of lipid droplet deposition.^56^ Menin interacts with multiple transcription factors and is participated in a variety of cellular processes, including gene activation and repression.^57^ Consistent with this concept, the crystal structure shows that menin contains a deep pocket that interacts similarly with MLL1 and JUND, suggesting that the multiple functions may be largely attributable to menin's key role as a core scaffold protein.^57^ Menin/MLL complexes have histone methyltransferase activity specific for H3K4me3 and play a critical role in tumourigenesis,^25^ hyperglycaemia^58^ and insulin resistance,^59^ but the putative biological function of menin/MLL1‐mediated H3K4me3 in renal fibrosis remains unknown. Our ChIP‐seq data analysis revealed that menin and H3K4me3 are extensively coenriched at many EMT driver, such as *Has2*, *Snai1*, *Twist1* and *Zeb2*, in the kidney tissues. These data indicate that the biological relevance of menin/MLL1‐mediated H3K4me3 in renal fibrosis. Importantly, SP2509, an activator of chromatin H3K4me3 modification,^44^ effectively rescued the suppression of E‐cadherin expression and reduced the elevation in N‐cadherin and α‐SMA expression induced by *MEN1* deficiency. Therefore, we propose that the special biological function of menin in preventing renal fibrogenesis at least partly depends on the epigenetic regulator activity of MLL.

The previous studies have reported that HGF plays a vital role in the mesenchymal‐to‐epithelial transition and the progression of renal fibrosis.^60^ Yang et al. indicated that HGF preserved the tubular epithelial cell phenotype by inhibiting the activation of myofibroblast.^29^ Our findings reveal that deficiency of *MEN1* resulted in a conspicuous diminish in HGF and Adamts5 mRNA and protein expression, whereas *MEN1* overexpression enhanced the expression of HGF and Adamts5. HGF has been evaluated for potential beneficial effects to protect against damage.^61^ High expression of HGF in kidneys reduced injury, leucocyte infiltrate and TGF‐β1 expression after acute ischaemic injury.^62^ HGF exerts antifibrotic effects at least partly by increasing matrix metalloproteinase (MMP) expression and reducing the expression of inhibitors of MMPs.^63^ Adamts5, a family of metalloproteinases, plays a critical role in blocking EMT signalling in gastric cancer^64^ and preventing ECM accumulation in mouse aortas,^30^ but the potential biological function of Adamts5 in the kidney remains elusive. We found that the KD of HGF by siRNA attenuated the expression of Adamts5, whereas exogenous HGF rescued the suppression of Adamts5 induced by *MEN1* deficiency. Importantly, injections of exogenous HGF dramatically resorted the expression of Adamts5 and ameliorated kidney fibrosis in the obstructed kidneys in the *Men1* ^Δ/Δ^ mice. Menin binds to the HGF and Adamts5 promoter loci and upregulates H3K4me3 and gene transcription, whereas TGF‐β treatment disrupted these functions. This study uncovered a previously unknown pathway for menin‐Hgf‐Adamts5, which is responsible for the progression of renal fibrosis. Notably, menin, HGF and Adamts5 are constitutively inactivated in the UUO‐induced TIF and patients with renal fibrosis, and they serve as critical renal fibrosis suppressors, suggesting the clinical significance of the menin‐HGF‐Adamts5 axis as a biomarker for the prognosis and assessment of renal fibrosis. These findings fully support that the administration of HGF is a therapeutic strategy for the chronic renal fibrosis with low menin expression.

The cell cycle is precisely controlled by multiple mechanisms, some of which menin‐regulated genes (such as p53, CHK1 and CHK2) that contribute to G2/M cell cycle progression.^25^, ^65^ Previous studies have demonstrated that cell cycle G2/M arrest in RTECs results in renal fibrosis^23^ and EMT plays a pivotal role in G2/M arrest of the cell cycle and parenchymal damage in renal fibrosis.^66^ However, menin has not been confirmed as a key regulator of cell cycle progression in the pathogenesis of renal fibrosis. Our results indicated that the disruption of *MEN1* by shRNA or sgRNA sensitized RTECs to G2/M arrest, which is consistent with an obvious enhance in the levels of p‐CHK2, profibrogenic genes, cyclin B1 expression and collagen 1 content. Notably, CM from the *MEN1*‐KO HK‐2 cells exposed to AA enhanced the proliferation of fibroblasts, which is a major feature of fibroblast activation during renal fibrogenesis.^67^ Based on these findings, we propose that there exists a signalling pathway and that *MEN1* in the RTECs may delay the progression of renal fibrosis by abating G2/M arrest of cell cycle. Our data also demonstrate that JNK signalling, known to promote TGF‐β and connective tissue growth factor gene transcription and initiate fibrosis,^68^ is activated during cell cycle G2/M arrest in *MEN1*‐deficient HK‐2 cells. These findings show that signals from kidney damage reduce the expression of the menin/MLL1 complex, thereby activating diverse signalling pathways involved in renal fibrosis (summarized in Figure 8O).

**FIGURE 9:**
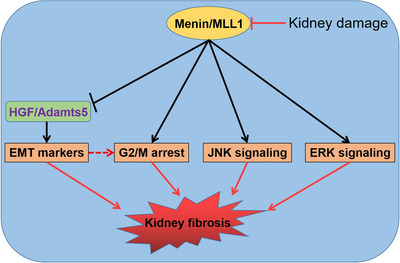
A schematic that summarizes the flow of signals from initial kidney damage to the suppression of menin/MLL1 expression, the expression of HGF‐Adamts5 and the activation of multiple signalling pathways involved in kidney fibrosis

In summary, we demonstrated a direct causal link between *MEN1* expression in the kidney and fibrosis, providing evidence for a vital function of *MEN1* in protecting the renal parenchyma against fibrotic injury. *MEN1* is a natural host defence barrier that protects against kidney fibrosis by regulating multiple key events, such EMT, G2/M arrest and JNK signalling; this kidney protection is performed at least partly via an epigenetic activation mechanism by which menin/MLL1‐mediated H3K4me3 controls HGF and Adamts5. However, we note that the *Men1* ^Δ/Δ^ mice are a whole‐body KO animal in the present study, and these contributions of *MEN1* may exist in extrarenal tissues, including lung and liver.^16^, ^25^, ^69^ Our work also emphasizes that the menin/MLL1‐HGF‐Adamts5 axis may be a therapeutic target for renal fibrosis caused by the disease (Figure 9).

## CONFLICT OF INTEREST

The authors declare that they have no known competing financial interests or personal relationships that could have appeared to influence the work reported in this paper.

## Supporting information

Supplement Material

Supplement Material

Supplement Material

Supplement Material

Supplement Material

Supplement Material

Supplement Material

Supplement Material

Supplement Material

Supplement Material

Supplement Material

Supplement Material
