# Supplementary figures and images for: Loss of MEN1 leads to renal fibrosis and decreases HGF‐Adamts5 pathway activity via an epigenetic mechanism
Source: Clin Transl Med. 2022 Aug 15;12(8):e982. doi: 10.1002/ctm2.982 (PMC9377152; doi:10.1002/ctm2.982)

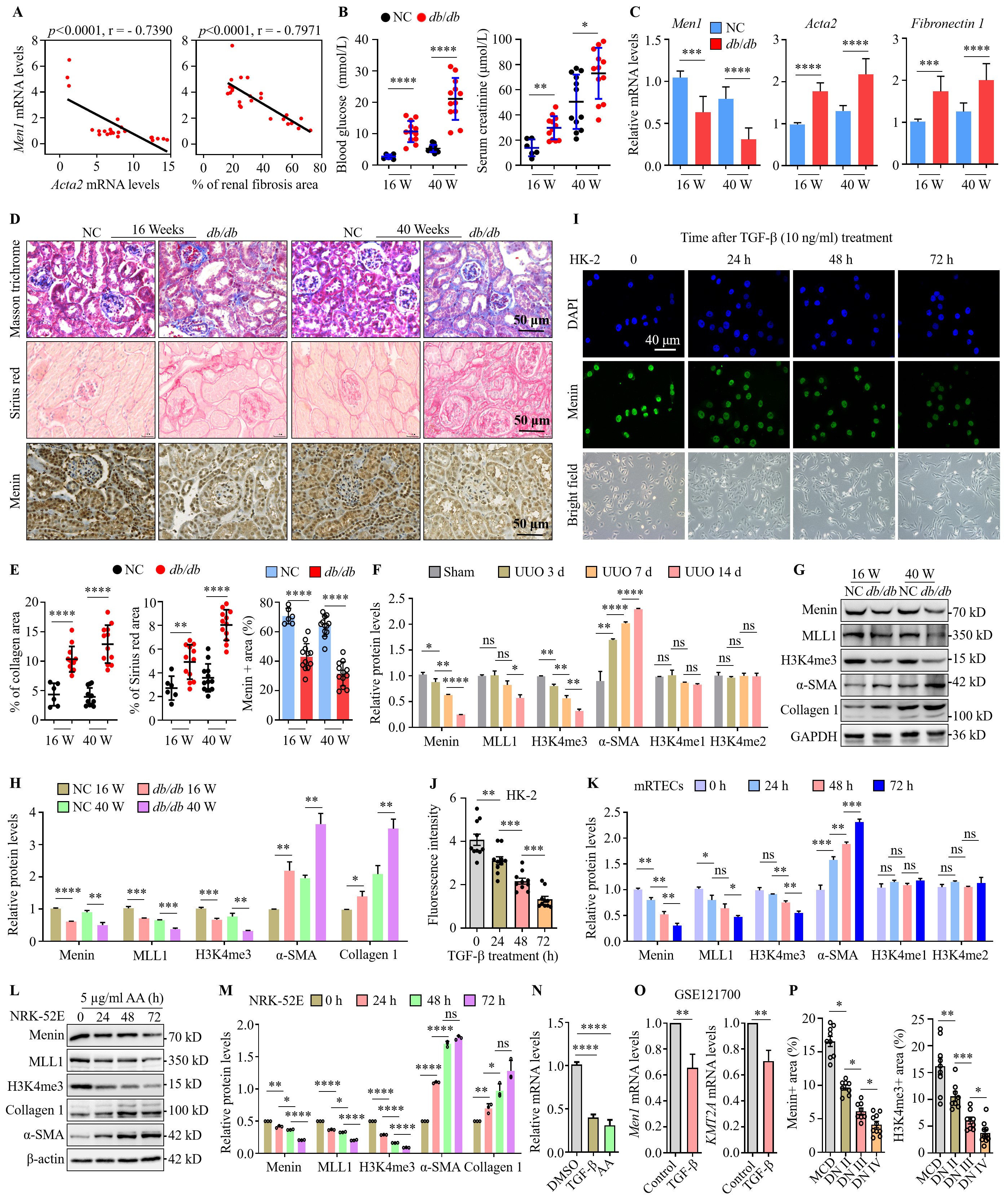

Supplement: Supplementary file 1 — Supplement Material [file CTM2-12-e982-s007.jpg]

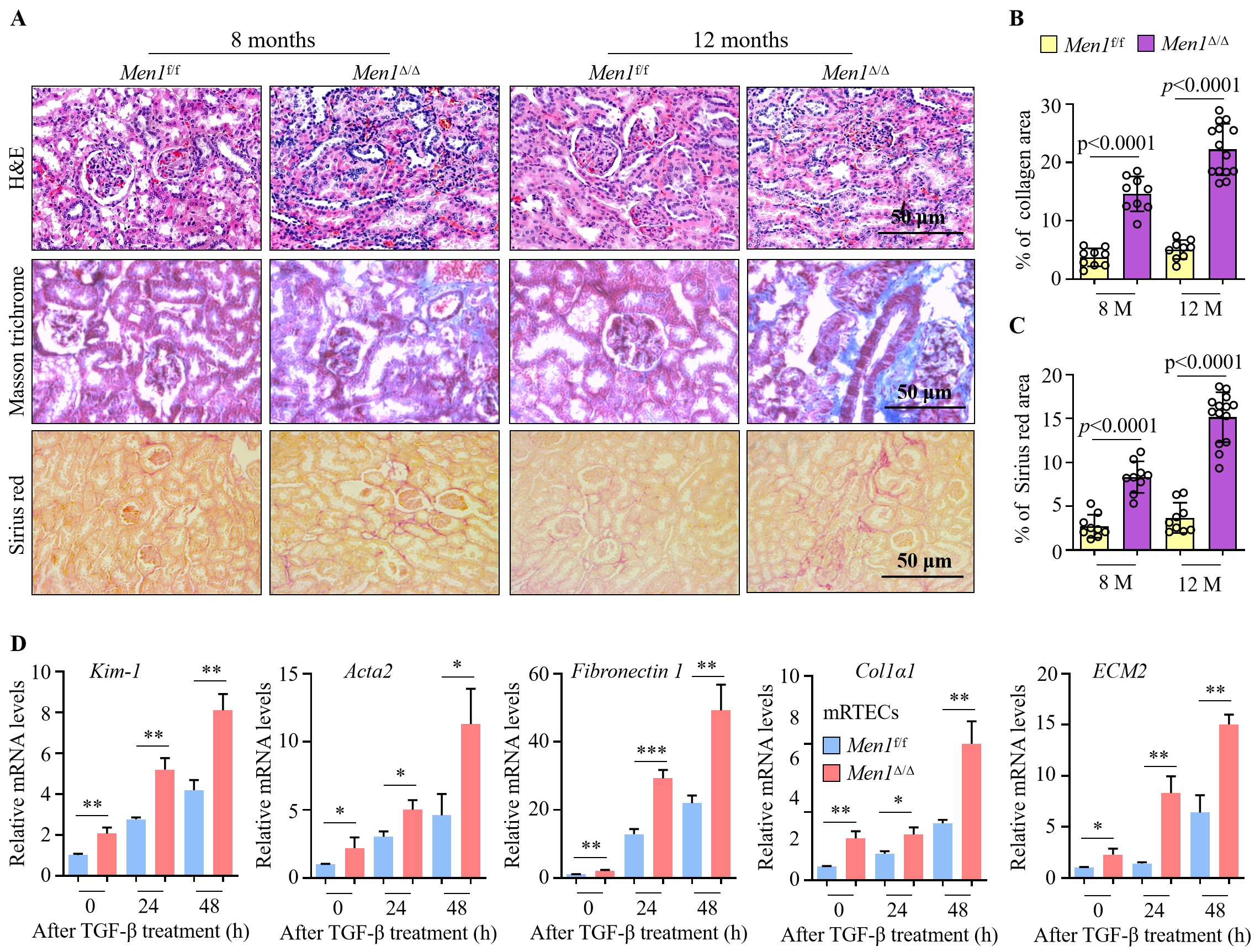

Supplement: Supplementary file 2 — Supplement Material [file CTM2-12-e982-s006.jpg]

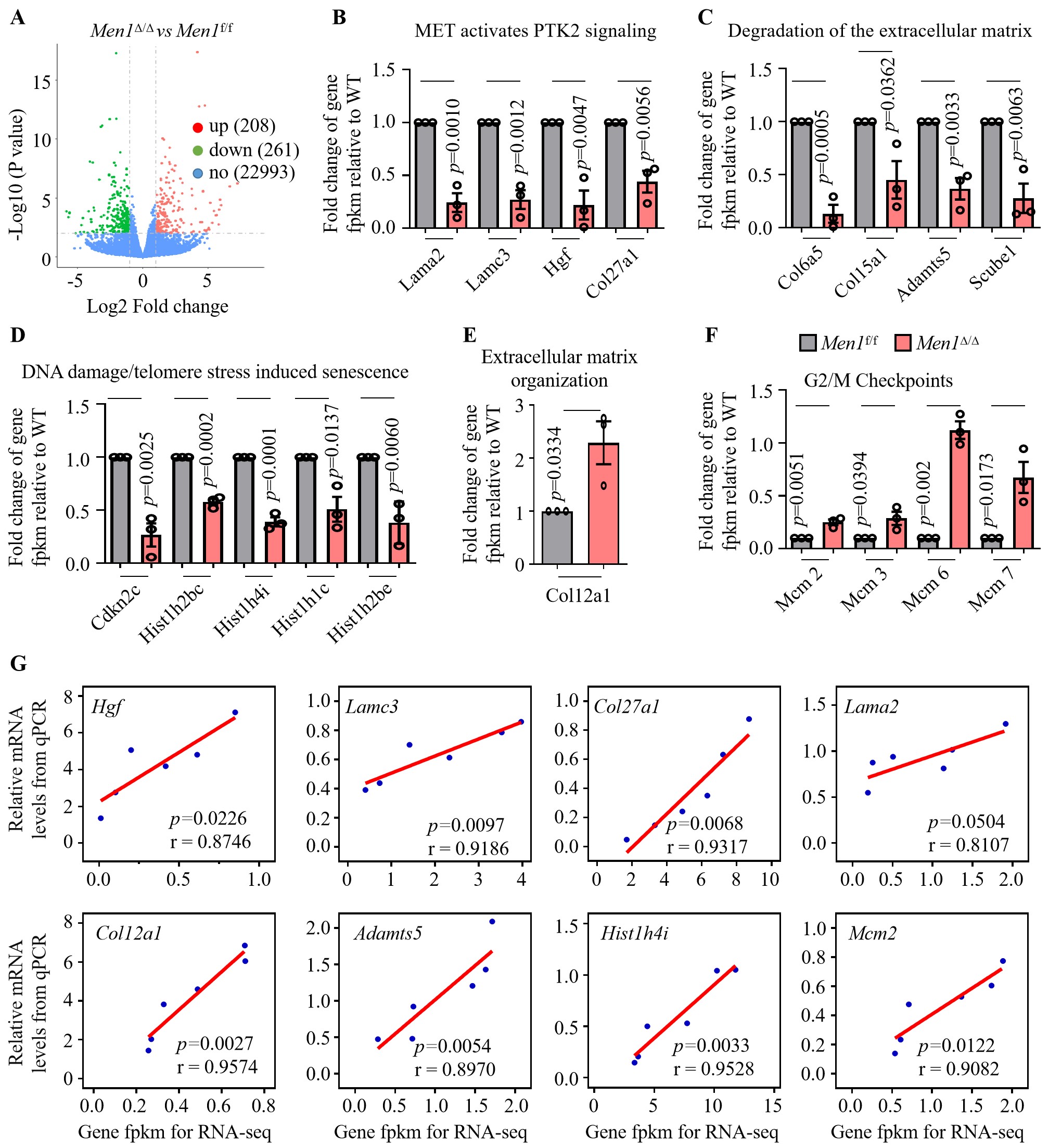

Supplement: Supplementary file 3 — Supplement Material [file CTM2-12-e982-s004.jpg]

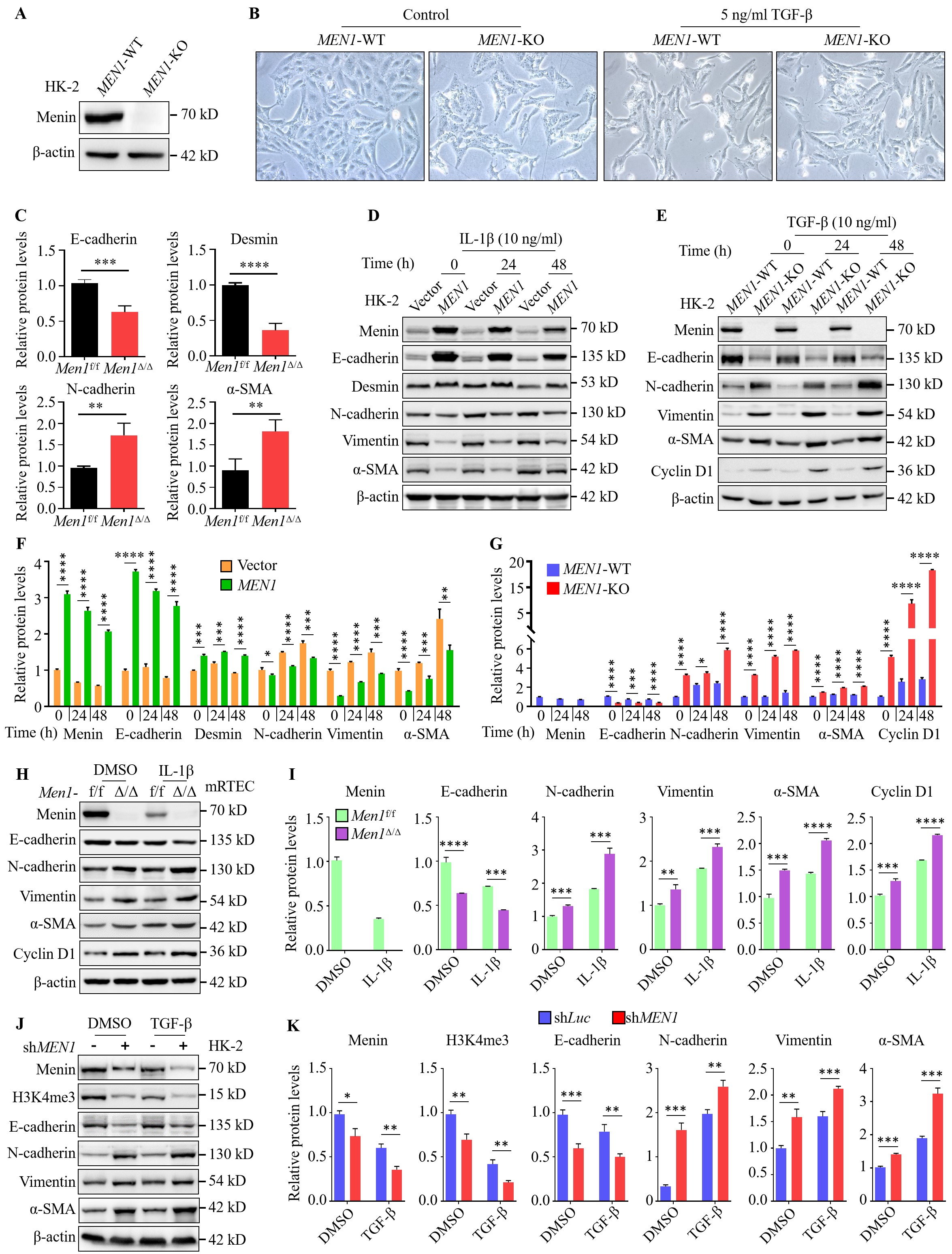

Supplement: Supplementary file 4 — Supplement Material [file CTM2-12-e982-s005.jpg]

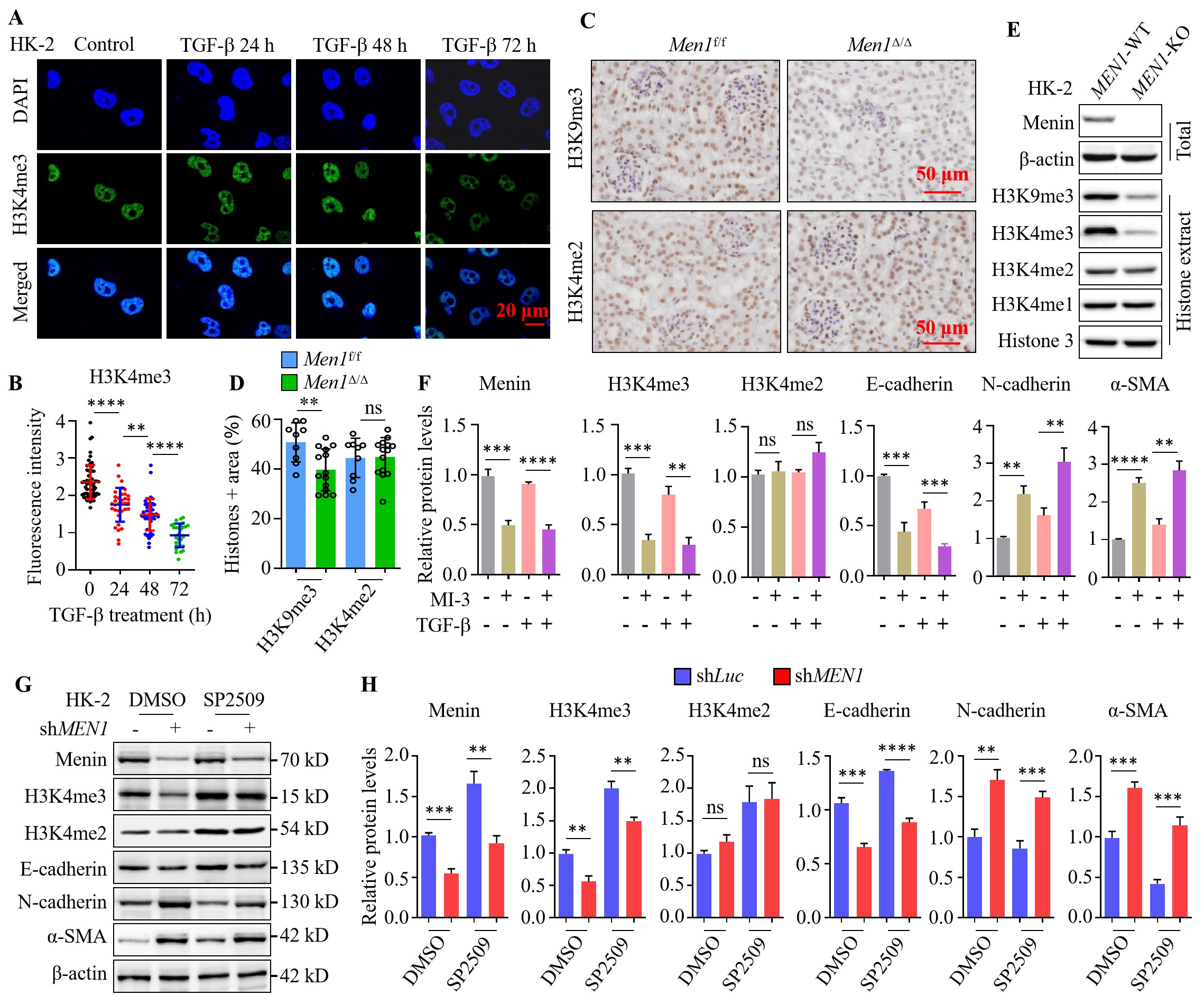

Supplement: Supplementary file 5 — Supplement Material [file CTM2-12-e982-s009.jpg]

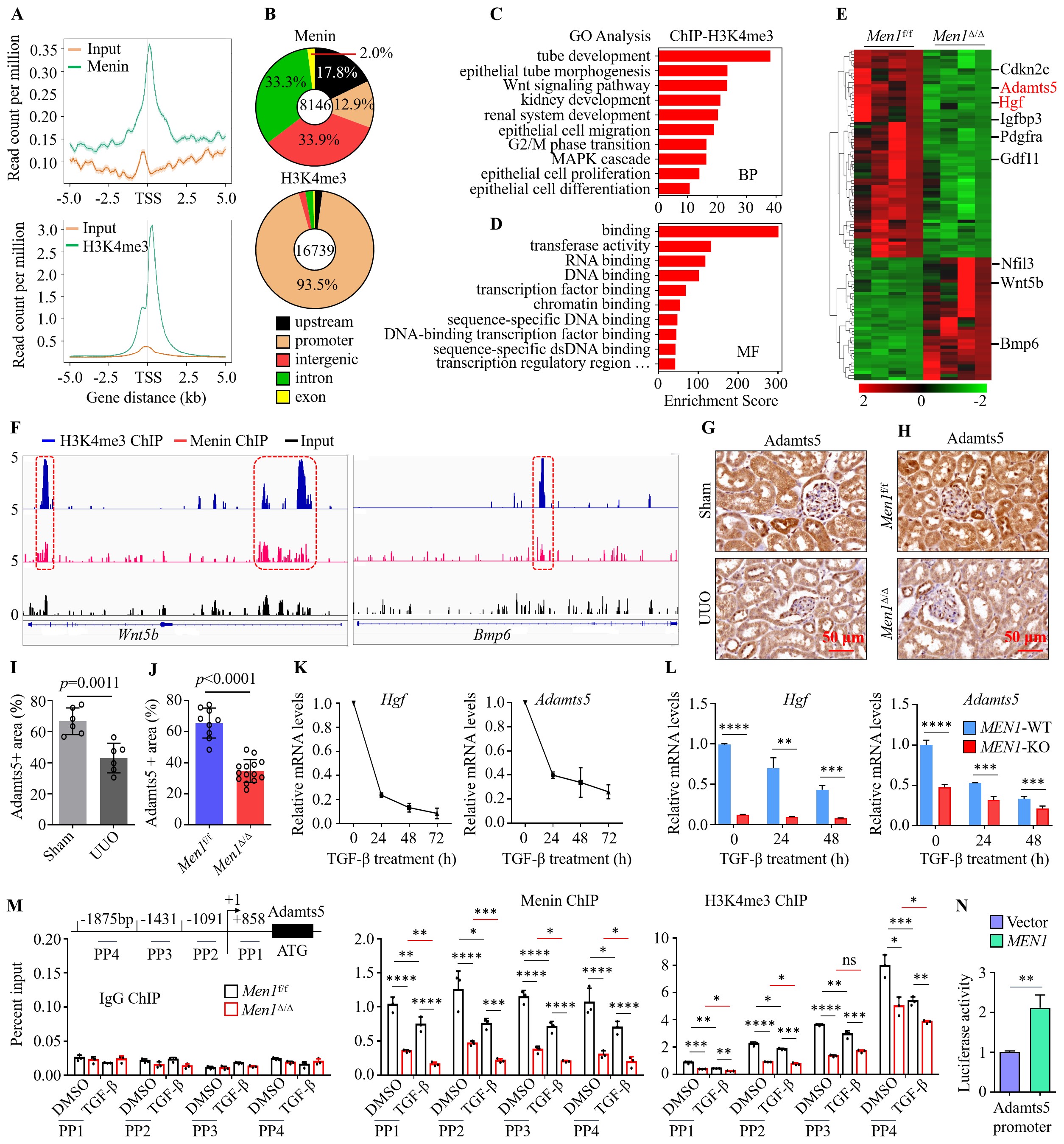

Supplement: Supplementary file 6 — Supplement Material [file CTM2-12-e982-s010.jpg]

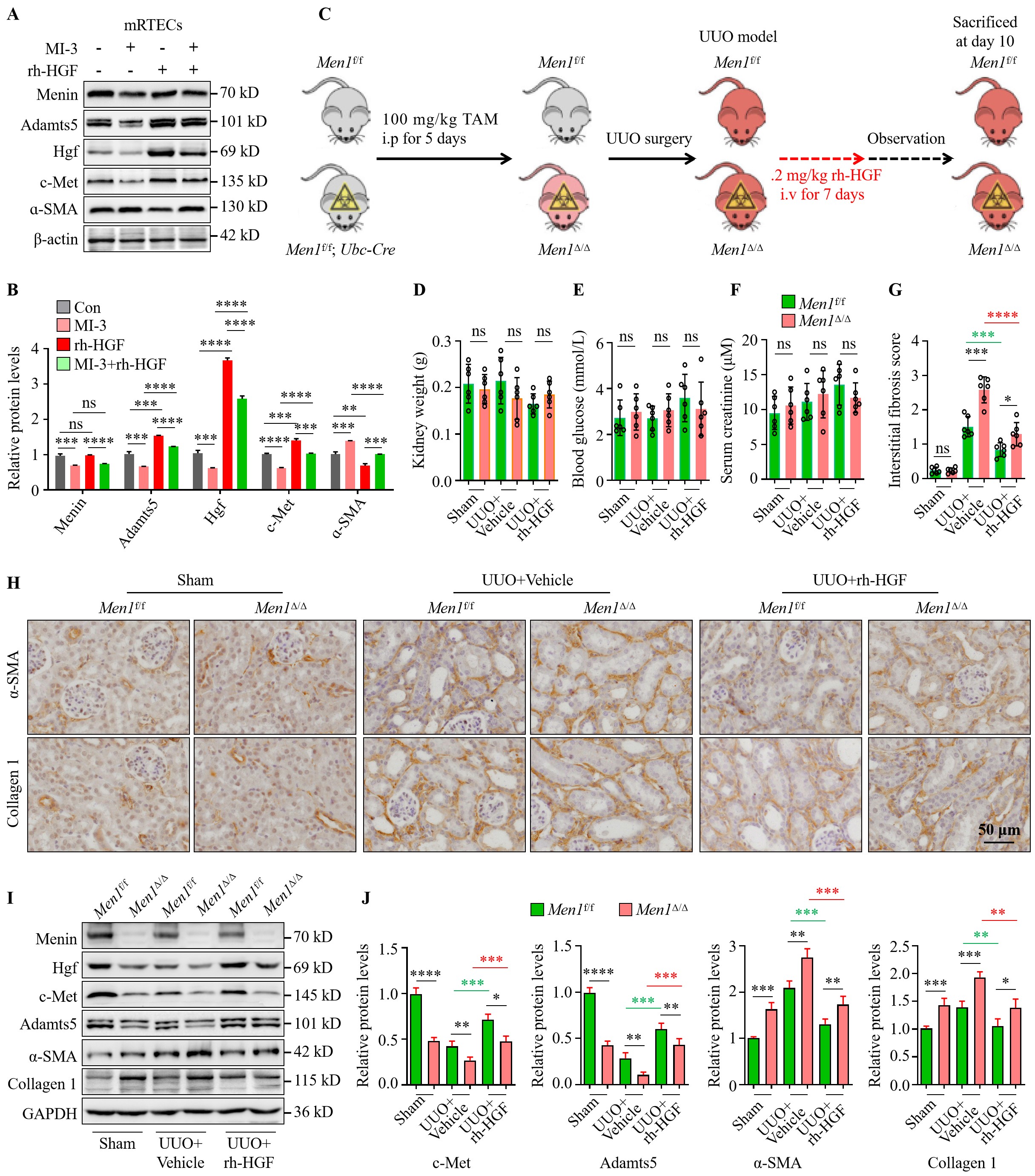

Supplement: Supplementary file 7 — Supplement Material [file CTM2-12-e982-s012.jpg]

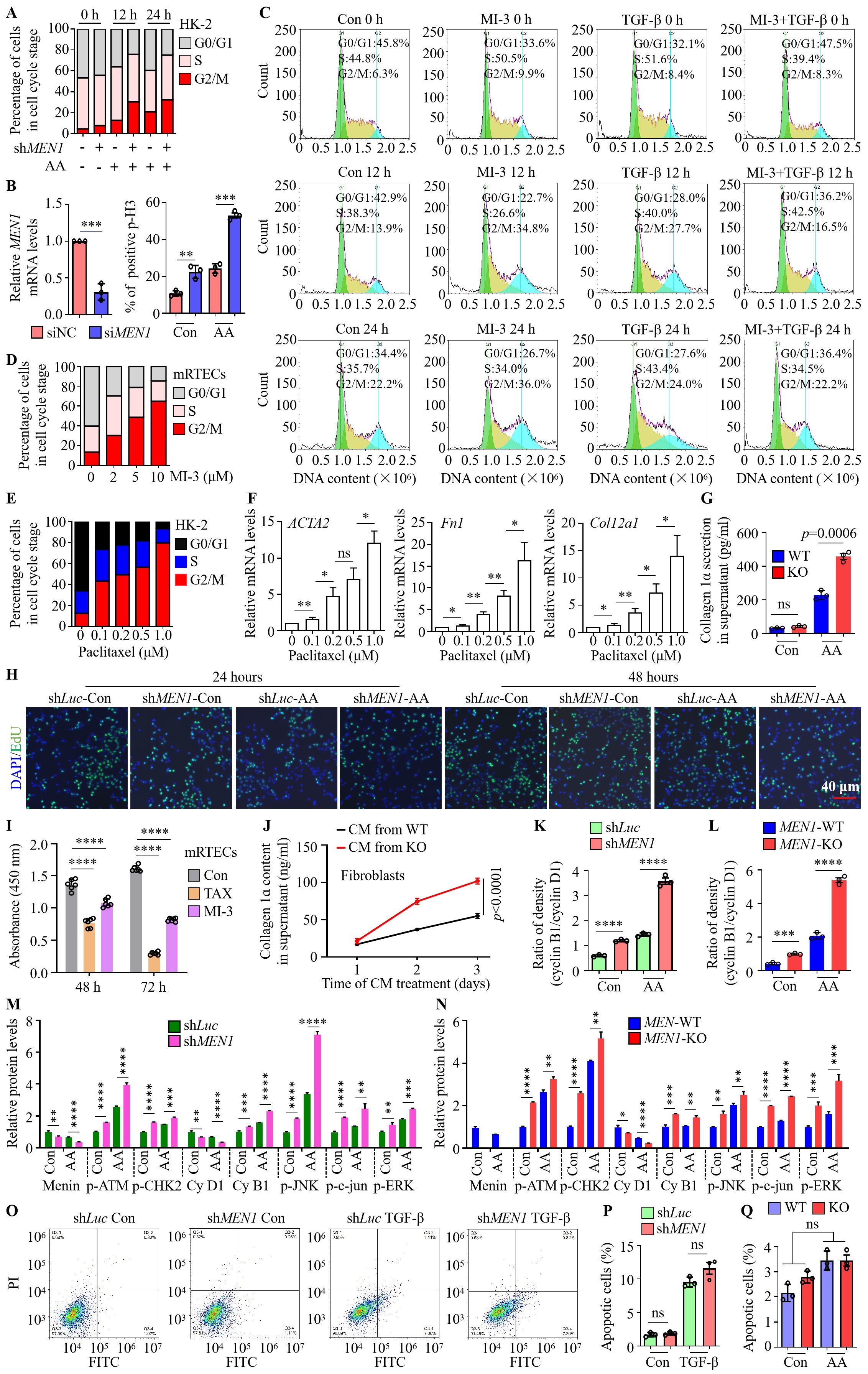

Supplement: Supplementary file 8 — Supplement Material [file CTM2-12-e982-s008.jpg]
