# Supplementary material for: Loss of MEN1 leads to renal fibrosis and decreases HGF‐Adamts5 pathway activity via an epigenetic mechanism
Source: Clin Transl Med. 2022 Aug 15;12(8):e982. doi: 10.1002/ctm2.982 (PMC9377152; doi:10.1002/ctm2.982)
Supplement: Supplementary file 9 — Supplement Material [file CTM2-12-e982-s001.docx]

Supplementary information

**Loss of *MEN1* leads to renal fibrosis and decreases HGF-Adamts5 pathway activity via an epigenetic mechanism**

Bangming Jin*, Jiamei Zhu, Yuxia Zhou, Li Liang, Yunqiao Yang, Tuo Zhang, Lifen Xu, Po Li, Ting Pan, Bing Guo*, Tengxiang Chen*, Haiyang Li*

*Corresponding author. Email: guobingbs@126.com；[BMJin@gmc.edu.cn](mailto:jinbangming052@yeah.net); [txch@gmc.edu.cn;](mailto:txch@gmc.edu.cn;) [lihaiyang@gmc.edu.cn](mailto:lihaiyang@gmc.edu.cn)


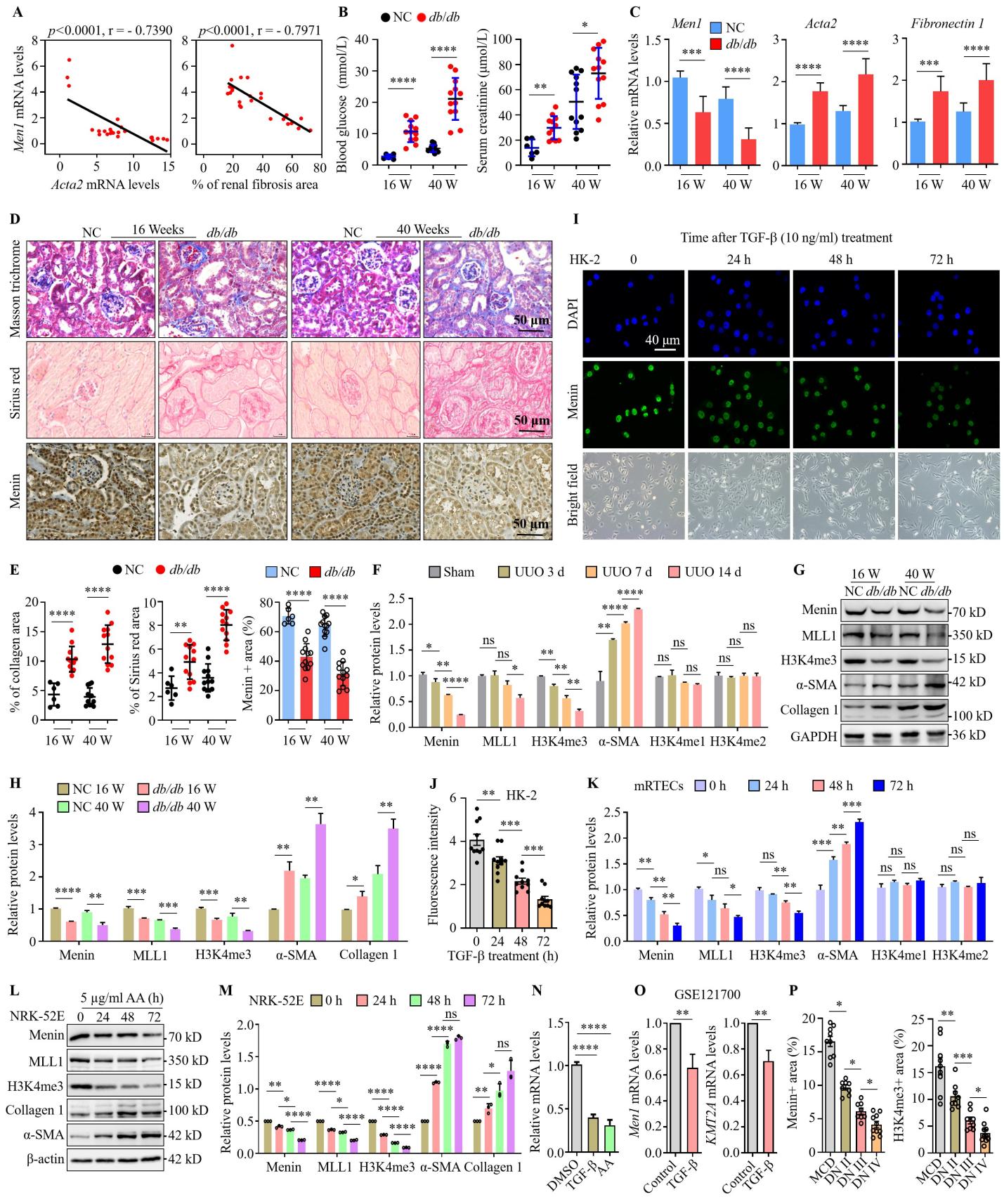


**Figure S1. Decreased *MEN1* expression in fibrotic kidney disease samples. (A)** Correlation analysis between the mRNA levels of *Men1* and mRNA levels of *Acta2* (left), as well as the renal fibrosis area (right); the Spearman correlation and *p* value by Spearman’s test are indicated and the data are represented as the mean±SD (n=24 mice in UUO group). (**B)** Quantification of the blood glucose and serum creatinine levels in normal control (NC) and *db*/*db* mice. (**C)** qPCR was used to detect the mRNA expression of indicated genes in the kidney tissues of the NC and *db*/*db* mice. (**D)** Representative images of Masson’s trichrome, Sirius red and menin IHC staining of kidney sections from the NC and *db*/*db* mice; scale bars 50 μm. (**E)** Quantification of the area of Masson’s trichrome, Sirius red and menin IHC staining in **D**. (**F)** Quantification of the grayscale image of the indicated proteins in **Figure 1F** (n=3 mice per group). **(G)** Western blotting was used to detect the expression of the indicated proteins in the kidney tissues of the NC and *db*/*db* mice. (**H)** Quantification of the grayscale image of indicated proteins in **G** (n=3 mice per group). (**I)** IF staining for menin (green) and DAPI (blue) in HK-2 cells at different times after exposure to 10 ng/ml TGF-β; scale bars 40 μm. (**J)** Quantification of the fluorescence intensity of menin in **I** (n=10 images per group). **(K)** Quantification of the grayscale image of the indicated proteins in **Figure 1G** (three biological replicates). (**L)** Western blotting was used to detect the expression of the indicated proteins in NRK-52E cells at the indicated time points after 5 µg/ml AA treatment. **(M)** Quantification of the grayscale image of the indicated proteins in **L** (three biological replicates). (**N)** qPCR was used to detect the mRNA expression of *Men1* in mRTECs treated with 10 ng/ml TGF-β or 5 µg/ml AA for 48 h. (**O)** Analysis of the mRNA expression of *Men1* and *KMT2A* in kidney tissues of the C57BL/6 mice with or without TGF-β. (**P)** Quantification of menin and H3K4me3 IHC staining in **Figure 1H.** n=6 mice in the NC and n=12 mice in the *db*/*db* group at 16 weeks; n=12 mice per group at 40 weeks. The data are represented as mean±SD; **p*<0.05, ***p*<0.01, ****p*<0.001, *****p*<0.0001.


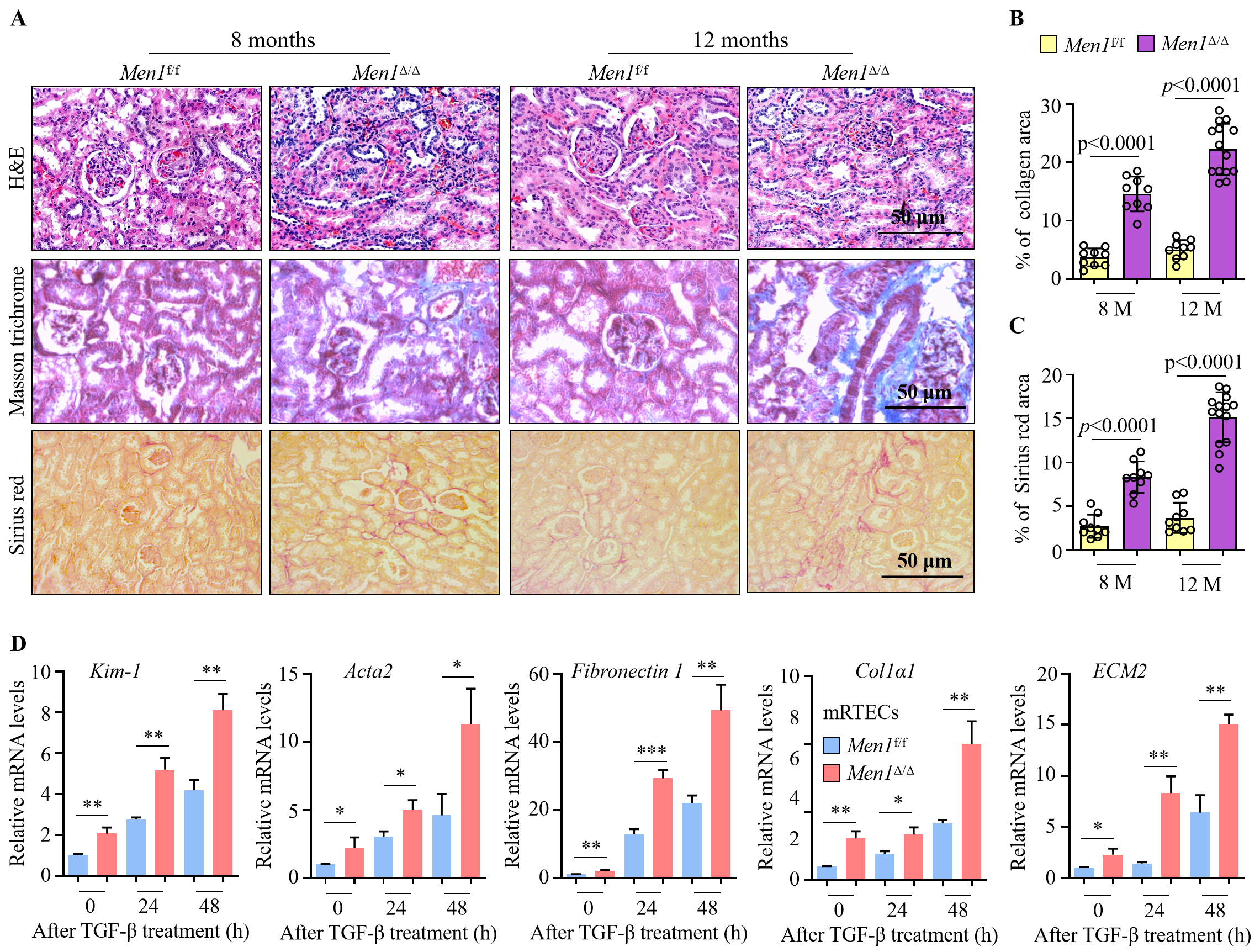


**Figure S2. Deletion of *Men1* results in progressive kidney damage and fibrosis. (A)** Representative images of H&E, Masson’s trichrome and Sirius red staining of kidney sections from the *Men1*^f/f^ and *Men1*^Δ/Δ^ mice at 8 and 12 months; scale bars 50 μm. **(B, C)** Quantification of the area of Masson’s trichrome and Sirius red staining in **A** (n=9 mice in the *Men1*^f/f^ and *Men1*^Δ/Δ^ groups at 8 months; n=9 mice in the *Men1*^f/f^ and n=14 mice in the *Men1*^Δ/Δ^ groups at 12 months). **(D)** qPCR was used to detect the mRNA expression of *Kim-1*, *Acta2*, *Fibronectin1*, *Col1α1*, and *ECM2* in the primary mRTECs at the indicated time points after 10 ng/ml TGF-β treatment (three biological replicates). The data are represented as mean±SD; **p*<0.05, ***p*<0.01, ****p*<0.001.


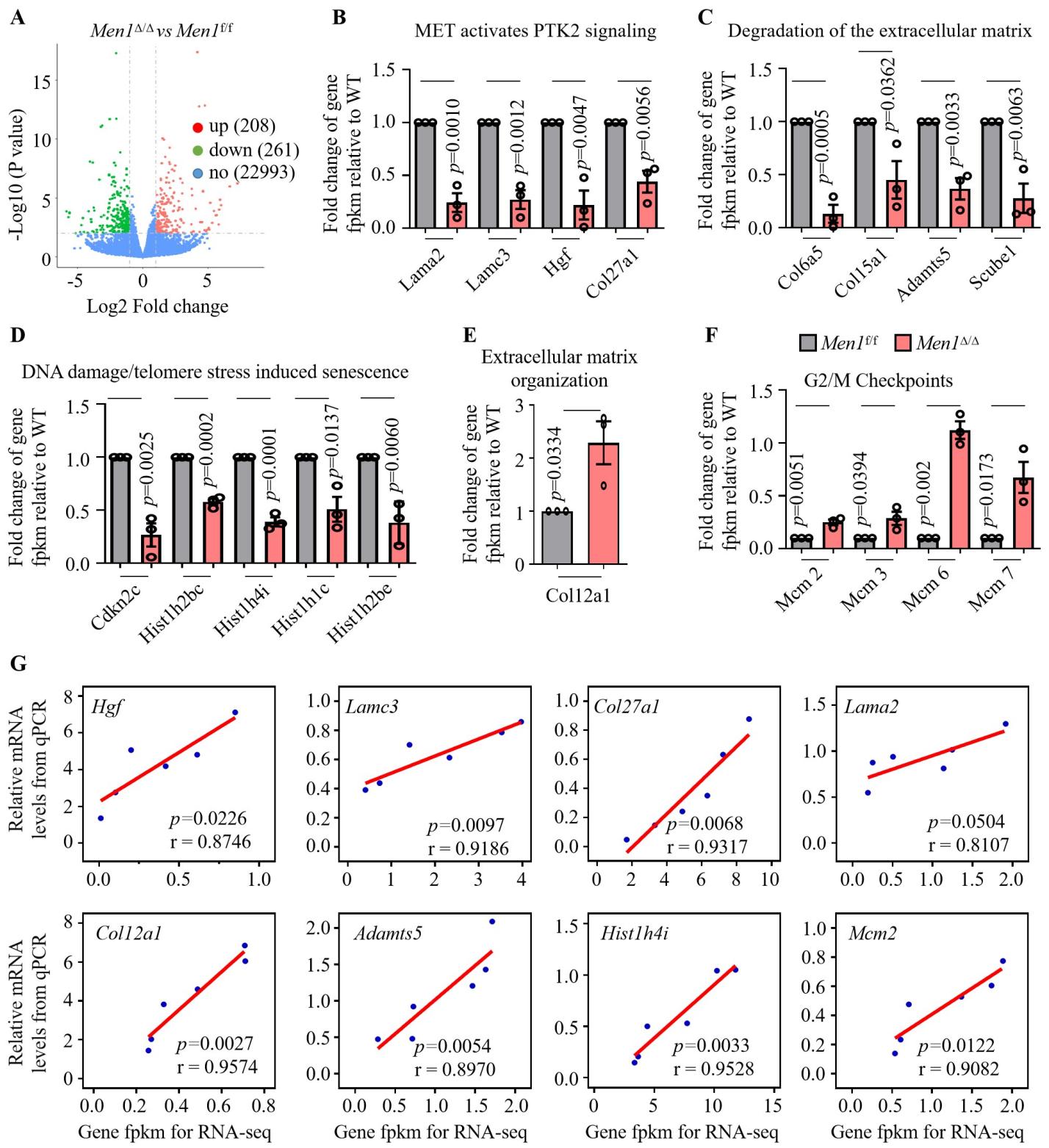


**Figure S3. Deletion of *Men1* induces fibrosis-related changes in signaling pathway networks. (A)** Volcano plots showing the expression profiles of mRNAs in the *Men1*^Δ/Δ^ mice compared with the *Men1*^f/f^ mice. (**B-F)** RNA-seq demonstrates that *Men1*^Δ/Δ^ mice have transcriptional patterns typically seen in fibrosis-related genes, including decreased levels of *Hgf*, *Col27a1*, *Adamts5,* and *Scube1*, as well as increased levels of in *Col12a1* and *Mcm2/3/6/7*. (**G)** RNA-seq vs qPCR correlation of gene expression measurement for the 8 selected candidates. Pearson r > 0.8 indicates a good level of correlation.


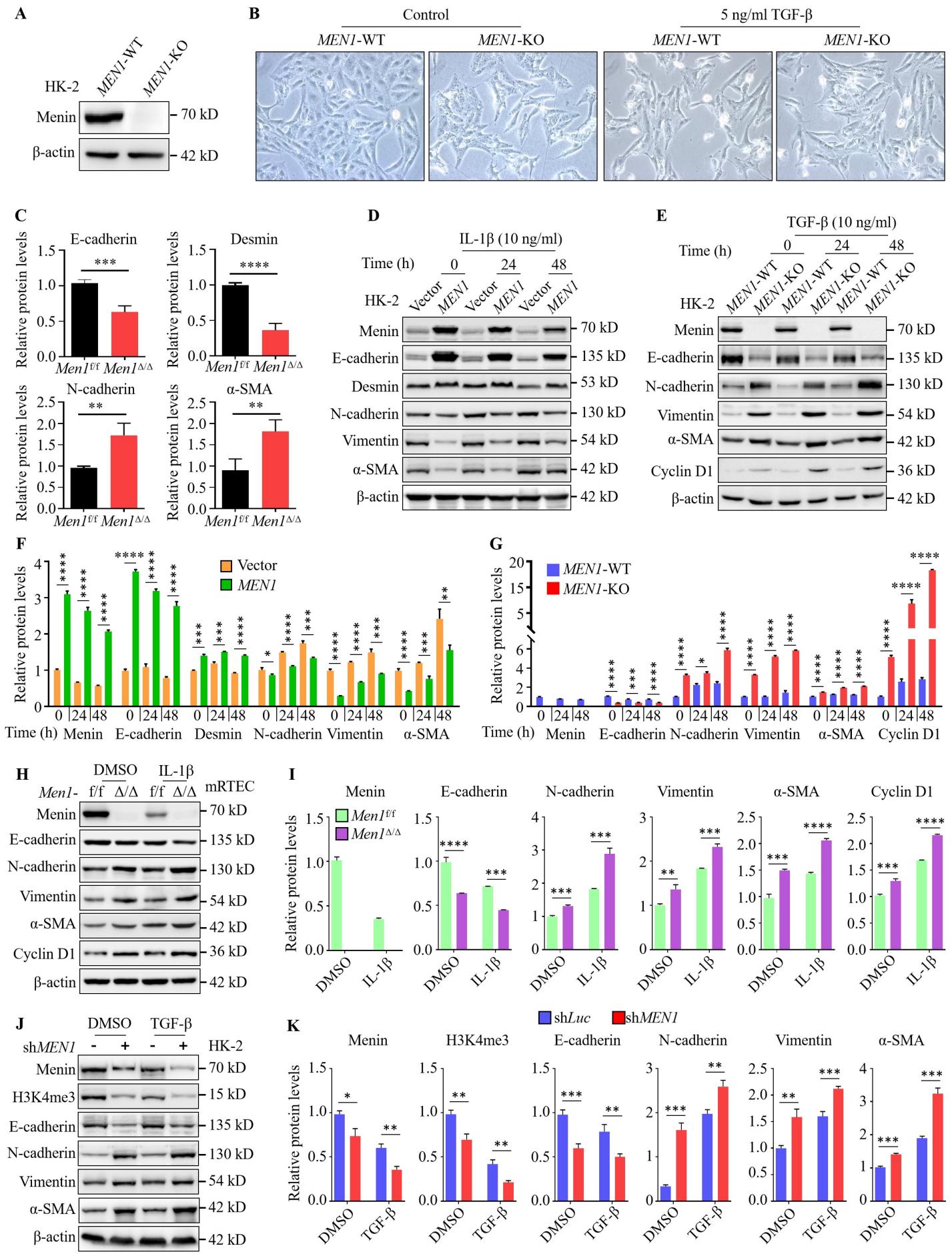


**Figure S4. Deletion of *Men1* induces tubular epithelial-to-mesenchymal transition.** (**A)** Western blotting was used to detect the expression of the menin protein in the *MEN1*-WT and *MEN1*-KO HK-2 cells. (**B)** Representative morphological image of the *MEN1*-WT and *MEN1*-KO HK-2 cells treated with 5 ng/ml TGF-β for 48 h. (**C)** Quantification of the grayscale image of the indicated proteins in **Figure 4E** (n=4 mice per group); the data are represented as mean±SD (*t*-test, two-sided). (**D)** Western blotting was used to detect the expression of the indicated proteins in the vector- and *MEN1*-HK-2 cells at the indicated time points after 10 ng/ml IL-1β treatment. (**E)** Western blotting was used to detect the expression of the indicated proteins in the *MEN1*-WT and *MEN1*-KO HK-2 cells at the indicated time points after 10 ng/ml TGF-β treatment. (**F)** Quantification of the grayscale image of the indicated proteins in **Figure 4F** (three biological replicates). (**G)** Quantification of the grayscale image of the indicated proteins in **E** (three biological replicates). **(H)** Western blotting was used to detect the expression of the indicated proteins in the *Men1*^f/f^ and *Men1*^Δ/Δ^ RTECs after 10 ng/ml IL-1β treatment. **(I)** Quantification of the grayscale image of the indicated proteins in **H** (three biological replicates). (**J)** Western blotting was used to detect the expression of the indicated proteins in the sh*Luc*- and sh*MEN1-* HK-2 cells after 10 ng/ml TGF-β treatment. (**K)** Quantification of the grayscale image of the indicated proteins in **J** (three biological replicates). The data are represented as mean±SD; **p*<0.05, ***p*<0.01, ****p*<0.001, *****p*<0.0001.


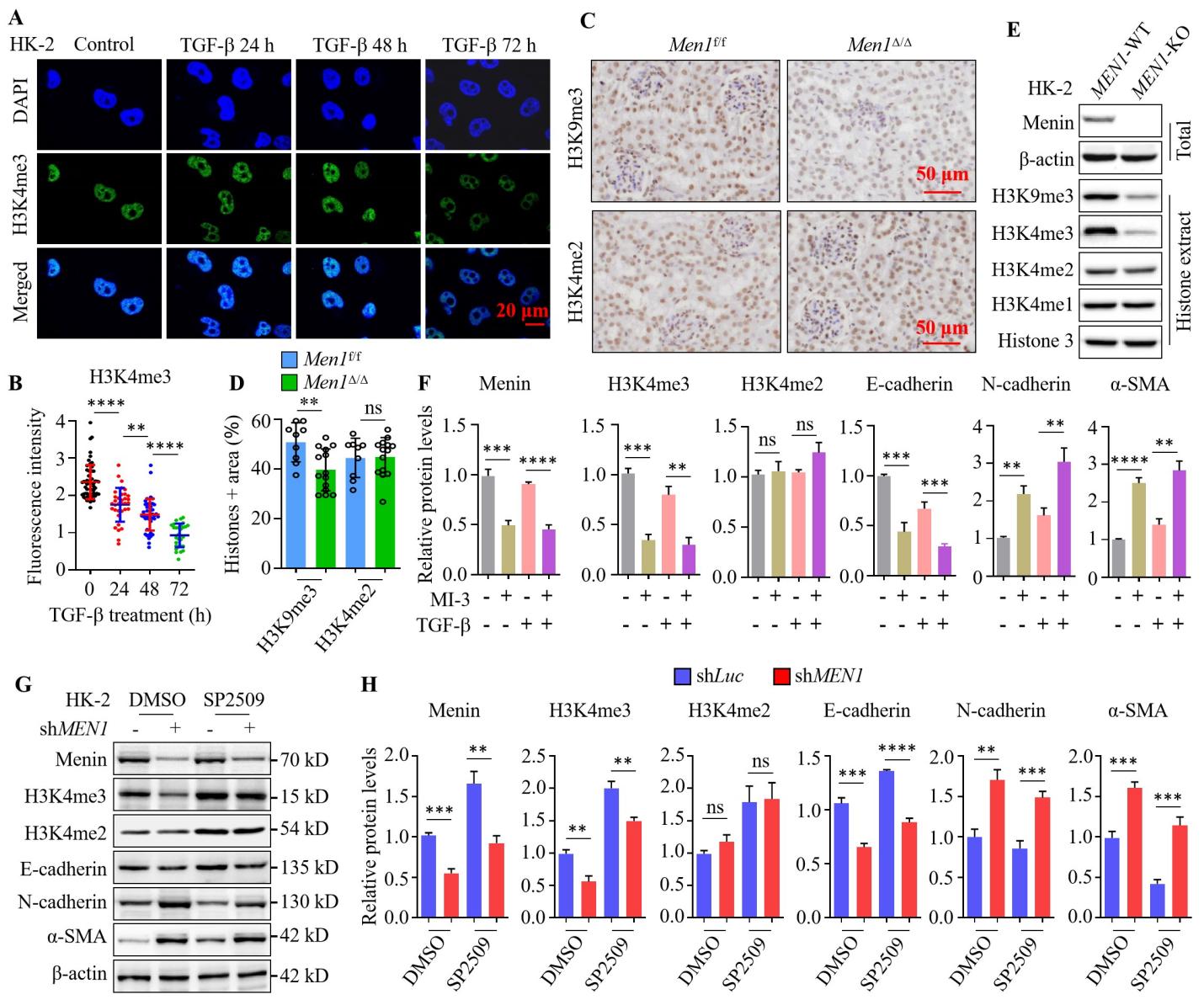


**Figure S5. Menin-dependent chromatin H3K4me3 modification is involved in regulating EMT. (A)** IF staining for H3K4me3 (green) and DAPI (blue) in HK-2 cells at the indicated time points after 2 ng/ml TGF-β treatment; scale bars 20 μm. (**B)** Quantification of H3K4me3 fluorescence intensity in **A**. **(C)** IHC staining for H3K9me3 and H3K4me2 in the kidney tissues of the *Men1*^f/f^ and *Men1*^Δ/Δ^ mice at 12 months; scale bars 50 μm. (**D)** Automatic quantification of H3K9me3 and H3K4me2 IHC staining in **C**. (**E)** Western blotting was used to detect the expression of the menin in total lysates and modification of the indicated histones in histone extracts of the *MEN1*-WT and *MEN1*-KO HK-2 cells. (**F)** Quantification of the grayscale image of the indicated proteins in **Figure 5F** (three biological replicates). (**G)** Western blotting was used to detect the expression of the indicated proteins in the sh*Luc*- and sh*MEN1*-HK-2 cells treated with 1 μM SP2509 for 48 h. (**H)** Quantification of the grayscale image of the indicated proteins in **G** (three biological replicates). The data are represented as mean±SD; **p*<0.05, ***p*<0.01, ****p*<0.001, *****p*<0.0001.


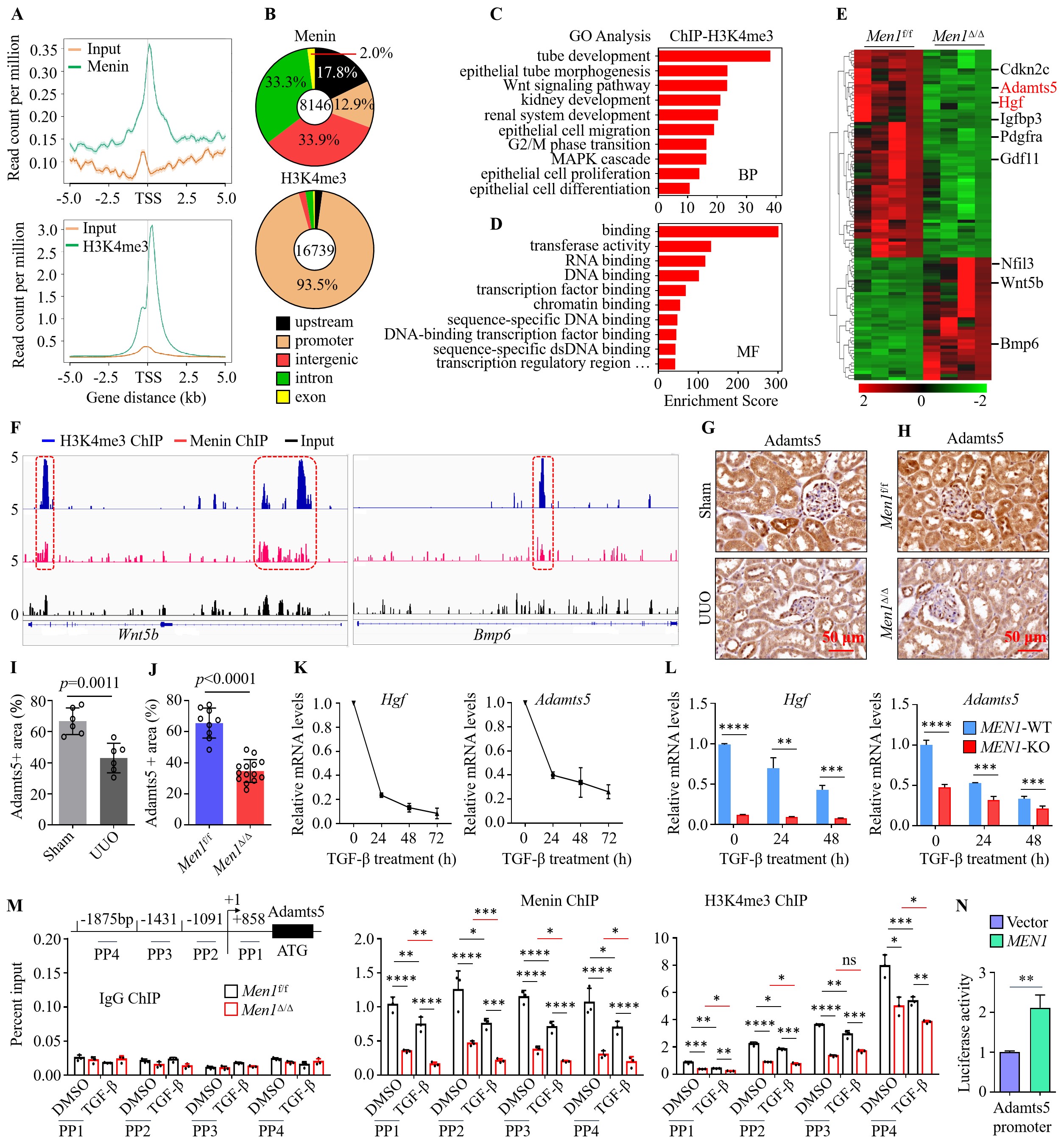


**Figure S6. Hgf/Adamts5 is a novel target gene that is epigenetically regulated by menin.** (**A)** Position of menin and H3K4me3 peak summits at the transcription start sites (TSSs) based on ChIP-seq analysis. (**B)** Genome-wide menin and H3K4me3 localization in different genome regions. (**C, D)** GO enrichment analysis showing representative biological processes (BPs) and molecular functions (MFs) for significantly enriched fibrosis- and nucleic acid binding-related genes based on the H3K4me3 ChIP-seq data. (**E)** Heatmap showing overlap of genes bound by menin and H3K4me3 with genes that are 39 genes with upregulated expression or 66 with downregulated expression in the RNA-seq data after *Men1* deletion. (**F)** Integrated genomics view of menin and H3K4me3 chromatin binding at the *Wnt5b* and *Bmp6* gene loci in kidney tissues of the *Men1*^f/f^ mice at 12 months. Gene bodies are schematically represented at the bottom of each track set. (**G**, **H)** IHC staining for Adamts5 in the kidney tissues of the sham and UUO mice 3 days after surgery, the *Men1*^f/f^ and *Men1*^Δ/Δ^ mice at 12 months; scale bars 50 μm. (**I**, **J)** Quantification of Adamts5 IHC staining in **G** (n=6 mice per group) and **H** (n=9 mice in the *Men1*^f/f^ and n=14 mice in the *Men1*^Δ/Δ^ group), respectively; the data are represented as the mean±SD (*t*-test, two-sided). (**K)** qPCR was used to detect the mRNA expression of the *Hgf* and *Adamts5* in primary mRTECs at the indicated time points after 10 ng/ml TGF-β treatment. (**L)** qPCR was used to detect the mRNA expression of the *Hgf* and *Adamts5* in the *MEN1*-WT and *MEN1*-KO HK-2 cells at the indicated time points after 10 ng/ml TGF-β treatment. (**M)** Schematic representation of the *Adamts5* gene promoter regions and primer pairs (PP) used for ChIP assays. ChIP-qPCR was performed with anti-menin or anti-H3K4me3 antibodies on samples from the immortalized *Men1*^f/f^ and *Men1*^Δ/Δ^ RTECs, and IgG served as the negative control (three biological replicates). (**N)** Impact of *MEN1* overexpression on *Adamts5* promoter activity in HK-2 cells by luciferase reporter analyses. The data are represented as mean±SD; **p*<0.05, ***p*<0.01, ****p*<0.001, *****p*<0.0001.


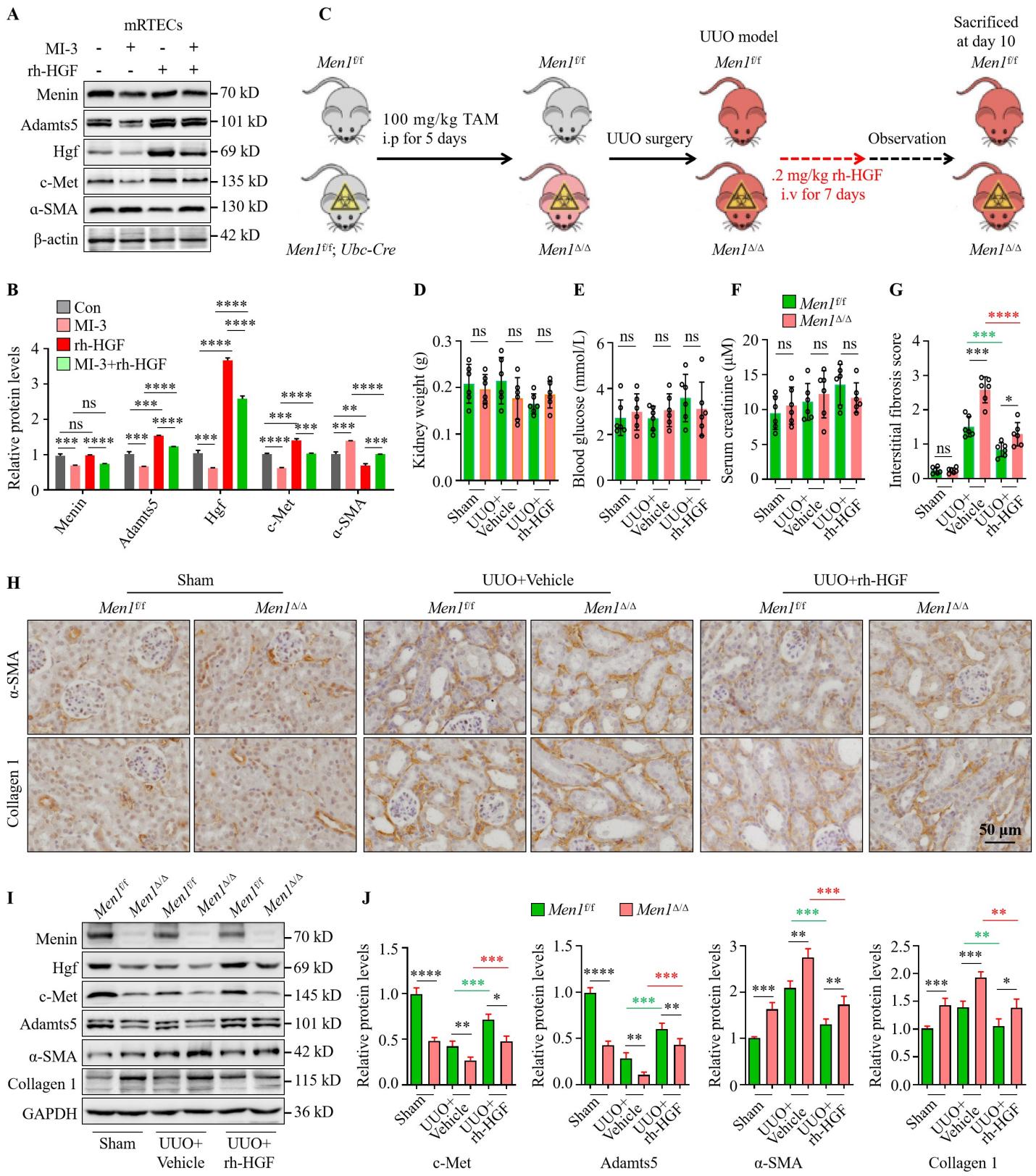


**Figure S7. Recombinant human HGF (rh-HGF) ameliorates renal fibrosis induced by *Men1* deletion.** **(A)** Western blotting was used to detect the expression of the indicated proteins in primary mRTECs 72 h after 10 μM MI-3 or 10 ng/ml rh-HGF alone or combined treatment. **(B)** Quantification of the grayscale image of the indicated proteins in **A** (three biological replicates). **(C)** Schematic representation of the drug studies performed *in vivo*. The *Men1*^f/f^ and *Men1*^f/f^; *Ubc-Cre* mice (6-8 weeks old) were induced by 100 mg/kg TAM for 5 days to obtain *Men1*^Δ/Δ^ mice; the *Men1*^f/f^ and *Men1*^Δ/Δ^ mice with renal fibrosis were established by UUO surgery and were randomly assigned to receive 200 μg/kg rh-HGF once one day for 7 days, and killed at Day 7 after treatment. **(D-F)** Physiological parameters including kidney weight, blood glucose, and serum creatinine were measured and quantified in the vehicle- and rh-HGF-treated *Men1*^f/f^ and *Men1*^Δ/Δ^ mice. **(G)** Quantitative analysis of interstitial fibrosis score in the obstructed kidneys of the vehicle- and rh-HGF-treated *Men1*^f/f^ and *Men1*^Δ/Δ^ mice. n=6 mice per group. **(H)** IHC staining for α-SMA and Collagen 1 in kidney sections from the vehicle- and rh-HGF-treated *Men1*^f/f^ and *Men1*^Δ/Δ^ mice; scale bars 50 μm; n=6 mice per group. **(I)** Western blotting was used to detect the expression of the indicated proteins in kidney tissues of the vehicle- and rh-HGF-treated *Men1*^f/f^ and *Men1*^Δ/Δ^ mice. **(J)** Quantification of the grayscale image of the indicated proteins in **I** (n=4 per group). The data are represented as mean±SD; **p*<0.05, ***p*<0.01, ****p*<0.001, *****p*<0.0001.


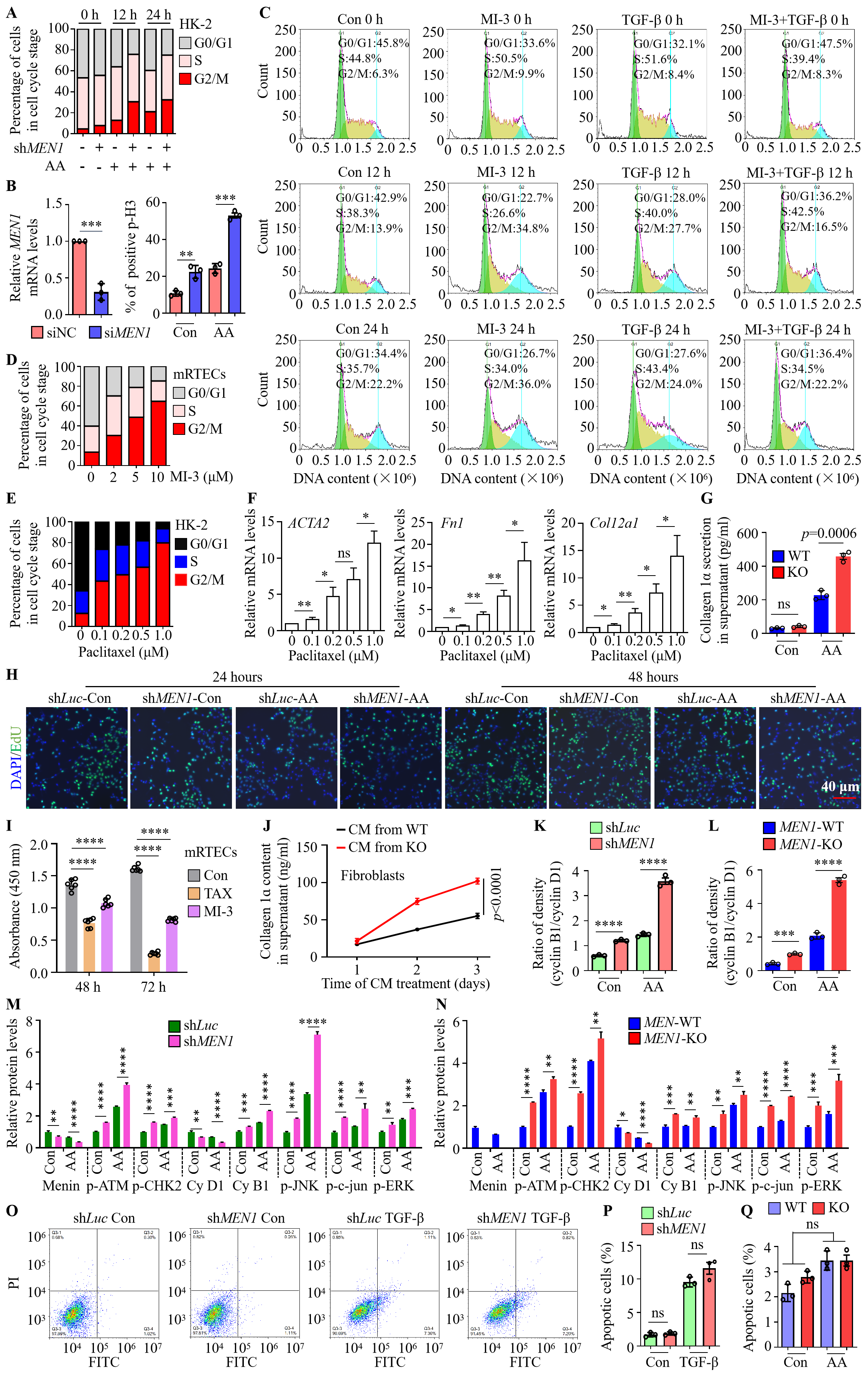


**Figure S8. Deletion of *MEN1* results in G2/M arrest and JNK signaling pathway activation. (A)** Cell cycle distribution in the sh*Luc*- and sh*MEN1*-HK-2 cells treated with 5 μg/ml aristolochic acid (AA) for 48 h. (**B)** qPCR was used to detect the mRNA expression of *MEN1* in the HK-2 cells transfected with siRNA for 48 h (left) and quantification of p-H3-positive cells in **Figure 8C** (right) (three biological replicates). (**C)** Cell cycle analysis by propidium iodide staining and flow cytometry in primary mRTECs at the indicated time points after 10 μM MI-3 or 10 ng/ml TGF-β alone or combined treatment. (**D)** Cell cycle distribution in primary mRTECs treated with different doses of MI-3 (0, 2, 5, 10 μM) for 72 h. (**E)** Cell cycle distribution in HK-2 cells treated with different doses of paclitaxel (0, 0.1, 0.2, 0.5, 1.0 μM) for 24 h. (**F)** qPCR was used to detect the mRNA expression of the indicated genes in HK-2 cells treated with different doses of paclitaxel (0, 0.1, 0.2, 0.5, 1.0 μM) for 24 h. (**G)** ELISA were used to measure the content of collagen 1α in the supernatant of the *MEN1*-WT and *MEN1*-KO HK-2 cells treated with 5 μg/ml AA for 48 h. (**H)** EdU (green) and DAPI (blue) staining were performed in sh*Luc*- and sh*MEN1-*HK-2 cells treated with 5 μg/ml AA for 24 and 48 h, scale bars 40 μm. (**I)** CCK-8 method was used to determine the proliferation of mRTECs treated with 10 μM MI-3 or 0.5 μM paclitaxel (TAX) for 48 and 72 h (three biological replicates). (**J)** Fibroblasts incubated with conditioned medium (CM) from the *MEN1*-WT and *MEN1*-KO HK-2 cells treated with 5 μg/ml AA, and ELISA were used to detect the content of collagen 1α in the supernatant. (**K, L)** Quantification of the ratio of cyclin B1 to cyclin D1 densities in **Figure 8M** and **8N** (three biological replicates), respectively. (**M)** Quantification of the grayscale image of the indicated proteins in **Figure 8M** (three biological replicates). **(N)** Quantification of the grayscale image of the indicated proteins in **Figure 8N** (three biological replicates). **(O)** Apoptosis as measured by Annexin V, propidium iodide staining and flow cytometry in the sh*Luc*- and sh*MEN1-*HK-2 cells treated with 10 ng/ml TGF-β treatment for 72 h. (**P)** Quantification of the apoptosis ratio in the sh*Luc*- and sh*MEN1-*HK-2 cells treated with 10 ng/ml TGF-β for 72 h. (**Q)** Quantification of the apoptosis ratio in the *MEN1*-WT and *MEN1*-KO HK-2 cells treated with 5 μg/ml AA for 24 h. The data are represented as mean±SD; **p*<0.05, ***p*<0.01, ****p*<0.001, *****p*<0.0001.
