# Supplementary material for: Loss of MEN1 leads to renal fibrosis and decreases HGF‐Adamts5 pathway activity via an epigenetic mechanism
Source: Clin Transl Med. 2022 Aug 15;12(8):e982. doi: 10.1002/ctm2.982 (PMC9377152; doi:10.1002/ctm2.982)
Supplement: Supplementary file 12 — Supplement Material [file CTM2-12-e982-s003.docx]

Supplementary information

**Loss of *MEN1* leads to renal fibrosis and decreases HGF-Adamts5 pathway activity via an epigenetic mechanism**

Bangming Jin*, Jiamei Zhu, Yuxia Zhou, Li Liang, Yunqiao Yang, Tuo Zhang, Lifen Xu, Po Li, Ting Pan, Bing Guo*, Tengxiang Chen*, Haiyang Li*

*Corresponding author. Email: guobingbs@126.com；[BMJin@gmc.edu.cn](mailto:jinbangming052@yeah.net); [txch@gmc.edu.cn;](mailto:txch@gmc.edu.cn;) [lihaiyang@gmc.edu.cn](mailto:lihaiyang@gmc.edu.cn)

**Table S1. The primers, siRNA, cell lines, plasmids, and mice used in the** **article**

| **RT-qPCR** |  |  |
| --- | --- | --- |
| M-*β-actin* | Forward | 5’-TGGTGTTTGTGGGAATGTC-3’ |
|  | Reverse | 5’-AGGCAGGAATAGTGGTCTC-3’ |
| M-*Men1* | Forward | 5’-GGAACTCTCTTCCCTCACATTC-3’ |
|  | Reverse | 5’-TCCTGCCTCAACTGTCTCTA-3’ |
| M-*Acat2* | Forward | 5’-CTGACAGAGGCACCACTGAA-3’ |
|  | Reverse | 5’-AGAGGCATAGAGGGACAGCA-3’ |
| M-*Fibronectin1* | Forward | 5’-ATGTGGACCCCTCCTGATAGT-3’ |
|  | Reverse | 5’-GCCCAGTGATTTCAGCAAAGG-3’ |
| M-*Kim-1* | Forward | 5’-AGAGGCATAGAGGGACAGCA-3’ |
|  | Reverse | 5’-AAGCAGAAGATGGGCATTGC-3’ |
| M-*ColIα1* | Forward | 5’-TGACTGGAAGAGCGGAGAGT-3’ |
|  | Reverse | 5’-GTTCGGGCTGATGTACCAGT-3’ |
| M-*Osteopontin* | Forward | 5’-GAAACCTGCTTCAGTGTGTCTG-3’ |
|  | Reverse | 5’-TTGAATTGCCACCATAAGTCTG-3’ |
| M-*Hgf* | Forward | 5’-GGCCAAGGAGAAGGTTACA-3’ |
|  | Reverse | 5’-GCATTTGAAGTTCTCGGGAG-3’ |
| M-*Adamts5* | Forward | 5’-GCATTGAGAACCACATCCG-3’ |
|  | Reverse | 5’-GCAAAAGTTCTTGAGGGTCG-3’ |
| M-*Lamc 3* | Forward | 5’-GAAGTTCAGCTCACATCGG-3’ |
|  | Reverse | 5’-GCACAGAATTCACAGAACTGG-3’ |
| M-*Col27a1* | Forward | 5’-GCAAGAAATTCACAGGACGG-3’ |
|  | Reverse | 5’-CTTGGTTGTCAGGTCTCTTG-3’ |
| M-*Lama2* | Forward | 5’-ACCTTGAATGCAGACTTGATG-3’ |
|  | Reverse | 5’-CCCGCCAACTGAAATATCC-3’ |
| M-*Col12a1* | Forward | 5’-CTACTGGTGAAACCTGCATCCA-3’ |
|  | Reverse | 5’-GGGCGCGGCTGTATGAG-3’ |
| M-*Hist1h4i* | Forward | 5’-AACATCCAGGGTATCACCAAG-3’ |
|  | Reverse | 5’-ACACCTTCAACACTCCGC-3’ |
| M-*Ecm2* | Forward | 5’-CAGCACATCAACCACTCTAC-3’ |
|  | Reverse | 5’-TCTCCTCTTATAGCATCCTCTTC-3’ |
| M-*Bax* | Forward | 5’-GGCGAATTGGAGATGAACTG-3’ |
|  | Reverse | 5’-TGCCATCAGCAAACATGTC-3’ |
| M-*Bcl2* | Forward | 5-’TGTGGATGACTGAGTACCTG-3’ |
|  | Reverse | 5’-GCCAGGAGAAATCAAACAGAG-3’ |
| M-*Aparf* | Forward | 5’-TCATTTGTAAGGACAGTGCTG-3’ |
|  | Reverse | 5’-GGTTCTCCATTCAGTTTCCAG-3’ |
| H-*β-actin* | Forward | 5’-TCAGAAGGATTCCTATGTGGGCGA-3’ |
|  | Reverse | 5’-TTTCTCCATGTCGTCCCAGTTGGT-3’ |
| H-*MEN1* | Forward | 5-’ATCACAGGCACCAAATTGGACAGC-3’ |
|  | Reverse | 5’-AACACTACCCAGGCATGATCCTCA-3’ |
| H-*Fibronectin1* | Forward | 5’-CACATTCCACAAGCGTCATG-3’ |
|  | Reverse | 5’-AGTCTCTGAATCCTGGCATTG-3’ |
| H-*Col12a1* | Forward | 5’-GAATCCATCTCCTAGTCCAGTG-3’ |
|  | Reverse | 5’-TGATCTGGTATTCTGTGTCTGC-3’ |
| H-*Col1a1* | Forward | 5’-GTGTTCCTGGAGACCTTGG-3’ |
|  | Reverse | 5’-CATCACCCTTAGCACCATCG-3’ |
| H-*ACTA2* | Forward | 5’-AGAGTTACGAGTTGCCTGATG-3’ |
|  | Reverse | 5’-ATGAAGGATGGCTGGAACAG-3’ |
| H-*Bax* | Forward | 5’-GCTGACATGTTTTCTGACGG-3’ |
|  | Reverse | 5’-GCCTTGAGCACCAGTTTG-3’ |
| H-*Bcl2* | Forward | 5’-GGATTGTGGCCTTCTTTGAG-3’ |
|  | Reverse | 5’-GGTACTCAGTCATCCACAGG-3’ |
| H-*Aparf* | Forward | 5’-CCAGAGGCTTCCACTTAATATTG-3’ |
|  | Reverse | 5’-GCTTTCAACACCCAAGAGTC-3’ |
| **ChIP** |  |  |
| M-*Hgf*-PP1 | Forward | 5’-CCCACGCTTTTCTTACCTG-3’ |
|  | Reverse | 5’-GGATGAACACTGACCCCA-3’ |
| M-*Hgf*-PP2 | Forward | 5’-TCATTAGCATGGTTCACAAGAG-3’ |
|  | Reverse | 5’-TCTGTGTTTCTGTTTGTCTCTT-3’ |
| M-*Hgf*-PP3 | Forward | 5’-GATGTCTGTGATTTCTAGGGC-3’ |
|  | Reverse | 5’-TGTCCTAGTTCTGCCTATTCC-3’ |
| M-*Hgf*-PP4 | Forward | 5’-TGTGTTGACAAAGATGGCC-3’ |
|  | Reverse | 5’-TGGGATAGGAGTATAGATCTTTGG-3’ |
| M-*Hgf*-PP5 | Forward | 5’-CCCACAGGACTATCTACAGC-3’ |
|  | Reverse | 5’-AGCCAAAGGATAAATCAGTCTG-3’ |
| M-*Adamts5*-PP1 | Forward | 5’-TTTTAGGTGTTTCCTTTGCTTG-3’ |
|  | Reverse | 5’-CGACCCACTTCCTTTCTTATTC-3’ |
| M-*Adamts5*-PP2 | Forward | 5’-TGAGGTAAGCCGAGGTTAG-3’ |
|  | Reverse | 5’-AGGGAGTAAGGGTGAGAAAG-3’ |
| M-*Adamts5*-PP3 | Forward | 5’-AGAGAACCAGGGAAAAGAGA-3’ |
|  | Reverse | 5’-CTGTTCTTGAGCGGGATATG-3’ |
| M-*Adamts5*-PP4 | Forward | 5’-TGTTTGCCAGAAAGTGTGAG-3’ |
|  | Reverse | 5’-GCCCACAATCTGTCACTTATC-3’ |
| **siRNA** |  |  |
| *MEN1* | Forward | 5’-GGAACCUGGCAGAUCUAGATT-3’ |
|  | Reverse | 5’-UCUAGAUCUGCCAGGUUCCTT-3’ |
| *HGF* | Forward | 5’-GCAAAGACUACCCUAAUCAAATT-3’ |
|  | Reverse | 5’-UUUGAUUAGGGUAGUCUUUGCTT-3' |
| NC | Forward | 5’-UUCUCCGAACGUGUCACGUTT-3’ |
|  | Reverse | 5’-ACGUGACACGUUCGGAGAATT-3’ |
| Sequencing primer | Forward | 5’-AAATGAGGGTTATTTGTTGGTGGG-3’ |
|  | Reverse | 5’-CTCTTCTGTCTTCCCTTCCTATGTG-3’ |
| **Genotype** |  |  |
| *Men1* flox | Wild type | 5’-TCCAGTCCCTCTTCAGCTTC-3’ |
|  | Mutant | 5’-GCCATTTCATTACCTCTTTCTCCG-3’ |
|  | Common | 5’-TACCACTGCAAAGGCCACGC-3’ |
| *Men1* deletion | Common | 5’-CCCACATCCAGTCCCTCTTCAGCT-3’ |
|  | Floxed | 5’-AAGGTACAGCAGAGGTCACAGAG-3’ |
|  | Deletion | 5’-GACAGGATTGGGAATTCTCTTTT-3’ |
| *UBC* cre | Transgene | 5’-GTGAAACAGCATTGCTGTCACTT-3’ |
|  | Forward | 5’-CTAGGCCACAGAATTGAAAGATCT-3’ |
|  | Reverse | 5’-GTAGGTGGA AATTCTAGCATCATC C-3’ |
| **Cell Lines** |  |  |
| Mouse: MEFs | This paper | N/A |
| Rattus: NRK-52E | ATCC | CRL-1571; RRID: CVCL_0468 |
| Mouse: RTEC | This paper | N/A |
| Human: HK-2 | ATCC | CRL-2190; RRID: CVCL_0302 |
| Human: GP2-293 | This paper | 631530 |
| Human: HEK-293 | ATCC | CRL-1573; RRID: CVCL_0045 |
| **Experimental Models: Organisms/Strains** |  |  |
| Mouse: C57 BL/KS *db*/*db* | Gempharmatech co.，Ltd | N/A |
| Mouse: C57BL/KS | Gempharmatech co.，Ltd | N/A |
| Mouse: *Men1*^f/f^:129S(FVB)-*Men1*^tm1.2Ctre^/J | Jackson Laboratory | 005109;RRID: IMSR_JAX:005109 |
| Mouse: *UBC*-cre: B6;129S-Tg(*UBC*-cre/ERT2)1Ejb/J | Jackson Laboratory | 007001;RRID: IMSR_JAX:007001 |
| **Plasmids and Virus Strains** |  |  |
| pLNCX2-*MEN1* vector | Takara | 631503 |
| Retrovirus-expressing sh*MEN1* | Takara | 631530 |
| pSpCas9-2A-GFP (PX458) vector | Addgene | 48138 |
| pSpCas9-2A-Puro (PX459) vector | Addgene | 48139 |
| pRL Renilla Luciferase Control Reporter Vectors | Promega | E2231 |
